# Association of malnutrition with cognitive frailty in China: a systematic review and meta-analysis

**DOI:** 10.3389/fpubh.2025.1567372

**Published:** 2025-04-14

**Authors:** Zhiren Zhu, Huiping Xue, Chunxia Huang, Jie Zhang, Jinheng Tu, Kenan Ling, Dongmei Gu

**Affiliations:** ^1^School of Nursing and Rehabilitation, Nantong University, Nantong, China; ^2^Nursing Department, Affiliated Hospital of Nantong University, Nantong, China

**Keywords:** cognitive frailty, malnutrition, older adult, China, systematic review, meta-analysis

## Abstract

**Background:**

With an aging population, China faces a growing burden of age-related health conditions, including cognitive frailty and malnutrition. This study aimed to investigate the current status of malnutrition in individuals with cognitive frailty in China and to assess the association between the two conditions.

**Methods:**

We conducted a comprehensive search of databases including PubMed, Web of Science, Scopus, Embase, Cochrane Library, CNKI, Wanfang, and Weipu up to April 26, 2024. Meta-analysis was performed using Stata/MP 16, with sensitivity and subgroup analyses to explore heterogeneity, and Begg’s and Egger’s tests to assess publication bias, applying the trim-and-fill method for correction.

**Results:**

Of 2,077 records, 19 were included. The pooled prevalence of cognitive frailty was 26% (95% confidence interval [CI]: 0.17–0.36, *p* < 0.01), and the prevalence of malnutrition was 45% (95% CI, 0.30–0.58, *p* < 0.01). A significant association was identified between cognitive frailty and malnutrition (odds ratio [OR] = 4.23, 95% CI: 2.56–6.99, *p* < 0.001), adjusted to OR = 3.00 (95% CI, 1.87–4.80) post-correction.

**Conclusion:**

Malnutrition is prevalent among individuals with cognitive frailty in China. Given its higher prevalence in community settings than in hospitals, early screening and specific interventions are crucial to address this issue.

## 1 Introduction

China has the highest number of older adults in the world (1). The National Bureau of Statistics of China reported that by the end of 2023, individuals aged ≥65 years reached 217.09 million, accounting for approximately 15.4% of the total population, an increase from 14.9% at the end of 2022 (2, 3). Incidences of chronic diseases, cognitive impairments, frailty, and malnutrition are on the rise, significantly affecting the aging population and contributing to increased prevalence and mortality across various healthcare domains (4).

Cognitive impairment and physical frailty are common in older adults. Studies have suggested a link between both conditions and malnutrition (a key connecting factor) (5–7). In 2013, the International Academy on Nutrition and Aging (IANA) and the International Association of Gerontology and Geriatrics (IAGG) introduced the novel concept of “Cognitive Frailty” (CF), which characterizes patients who exhibit physical frailty and cognitive impairment with a Clinical Dementia Rating (CDR) of 0.5, specifically excluding those with Alzheimer’s disease and other forms of dementia (8). Ruan et al. refined CF’s classification by proposing distinctions between reversible and potentially reversible CF (9). Recent research indicates that CF prevalence among older individuals in China is approximately 15%, underscoring its significant impact on individual health and societal resources (10).

Malnutrition is a global issue that includes traditional concerns, such as underweight, micronutrient deficiencies, and nutritional excesses related to overweight, obesity, and associated non-communicable diseases (11). This study primarily examined these traditional issues. Malnutrition negatively affects tissues, organs, body function, and clinical outcomes (12). In older adults, it is linked to cognitive impairment, physical frailty, depression, and increased risk of mortality, highlighting the need for targeted nutritional interventions in this population (13–16). Current data reveal that nearly half of older adults in China are either experiencing malnutrition or are at risk of malnutrition, with a combined prevalence rate of 41.2% (17). Owing to physical factors, older adults are prone to malnutrition (18, 19). Therefore, enhancing regular screening and assessment of nutritional status in older adults will help to promptly identify and address malnutrition.

Current research has demonstrated that malnutrition can cause further deterioration of CF in older adults (20, 21). CF occurrence and progression and malnutrition are influenced by multiple shared risk factors, including social factors associated with aging (e.g., sex, age, literacy, and poverty), physiological changes (e.g., reduced sense of smell and taste, reduced central and peripheral drive to eat, and delayed gastric emptying), and pathological conditions (e.g., depression, dementia, and somatic diseases) (22). Although studies have explored the relationship between CF and malnutrition, systematic reviews and meta-analyses specifically examining this association in the older Chinese population are lacking. Therefore, this study aimed to comprehensively synthesize the current literature on the status of malnutrition among patients with CF in China and to fill the research gap and inform clinical practices to improve the health of older adults in China.

## 2 Materials and methods

This systematic review and meta-analysis were conducted per the Preferred Reporting Items for Systematic Reviews and Meta-Analyses (PRISMA) guidelines (23). The protocol was registered in the International Prospective Register of Systematic Reviews (registration number: CRD42024538854).

### 2.1 Search strategy

The PubMed, Web of Science, Scopus, Embase, Cochrane Library, China National Knowledge Infrastructure (CNKI), Wanfang, and Weipu (VIP) databases were searched independently by two researchers for literatures published from the inception of each database through April 26, 2024. The following search terms were used: malnutrition, nutritional deficiency, nutritional deficiencies, undernutrition, malnourishment, malnourishments, frailty, cognitive frailty, cognitive decline, cognitive impairment, frailty*, and debilitating* (for detailed information, see the Supplementary Material).

### 2.2 Study selection

The inclusion criteria were (a) cross-sectional, cohort, or longitudinal studies; (b) sample population aged ≥60 years among Chinese; (c) articles that examined patients with cognitive impairment who were malnourished. These diagnoses are determined using various assessment tools. Physical frailty is evaluated using either the frailty phenotype (FP) (24) or the FRAIL scale (FS) (25). Cognitive impairment is assessed with the Montreal Cognitive Assessment (MoCA) (26), Mini Mental State Examination (MMSE) (27), Short Portable Mental Status Questionnaire (SPMSQ) (28) and Clinical Dementia Rating (CDR) (29); malnutrition or risk of malnutrition is determined using the following instruments: Mini Nutritional Assessment (MNA) (30), Nutritional Risk Screening 2002 (NRS-2002) (31), and Short-Form Mini Nutritional Assessment (MNA-SF) (32). (d) articles that reported the diagnostic criteria for both CF and malnutrition; and (e) articles in English and Chinese.

The exclusion criteria were (a) conference and review articles; (b) articles having patients with dementia or other conditions affecting CF diagnosis. (c) multiple publications from the same dataset, with only the most comprehensive or up-to-date selection; and (d) studies with no description of the diagnostic tool used for malnutrition.

### 2.3 Study selection and data extraction

The retrieved literature was imported into EndNote X9 (Clarivate, Philadelphia, PA, United States). Initially, two researchers removed duplicate studies and screened the articles based on the specified inclusion and exclusion criteria. Titles and abstracts that met the inclusion criteria were further assessed using full-text reviews to determine their inclusion eligibility for analysis. In case of disagreement during the screening process, the corresponding author of the paper was consulted.

Following screening, two researchers independently extracted and recorded data from the articles into an Excel spreadsheet, including the authors (publication year), study period, city or province, sample size, average age, percentage of female participants, CF assessment tools, diagnostic tools for malnutrition, criteria for defining malnutrition, and malnutrition prevalence among those with CF. Data extraction results were compared for consistency. Any discrepancies were resolved by consulting the corresponding authors.

### 2.4 Quality evaluation

Two researchers independently assessed all included articles, and any discrepancies were resolved by consulting a third expert in evidence-based practice associated with this paper. The cross-sectional study used the evaluation criteria recommended by the Agency (33), and the Agency for Healthcare Research and Quality (AHRQ) criteria included 11 items with “yes,” “no,” or “unclear” options, with only a “yes” response receiving one point. The results of the quality evaluation of each article are shown in the Supporting Information. It should be noted that the AHRQ system employs a binary yes/no approach, which may not capture subtle differences in methodological quality, thereby reducing its sensitivity to the true quality of the studies.

### 2.5 Statistical analysis

This study primarily investigated the prevalence and factors associated with malnutrition among patients with CF in China, with results presented using a 95% confidence interval (CI). Stata/MP 16 (StataCorp LLC, College Station, TX, United States) was used for statistical analyses in prevalence and risk factors.

The prevalence of CF among Chinese and malnutrition prevalence in patients with CF in China was defined as the ratio of the sum of the number cases (numerator) extracted from each article to the summed sample size (denominator) from each studies. Random-effects model was used to pool the prevalence estimates, and sensitivity analyses, along with begg’s tests, were performed to assess the results. When *p* < 0.05, the trim-and-fill methods was applied to adjust the prevalence estimates.

Heterogeneity among studies was evaluated using Cochran’s Q statistic, with *p* < 0.05 indicating significant heterogeneity. The I^2^ statistic was used to measure the extent of the heterogeneity. If I^2^ > 50%, a random-effects model was applied; if I^2^ < 50%, a fixed-effects model was used.

The results and characteristics of this study are presented as forest plots. Publication bias was analyzed using Egger’s and Begg’s tests, and significant bias was indicated in the results of both tests (*p* < 0.05), prompting further examination using the trim-and-fill method. To explore the potential sources of heterogeneity, sensitivity and subgroup analyses were conducted on prevalence and associations.

## 3 Results

### 3.1 Study selection

**Figure 1:**
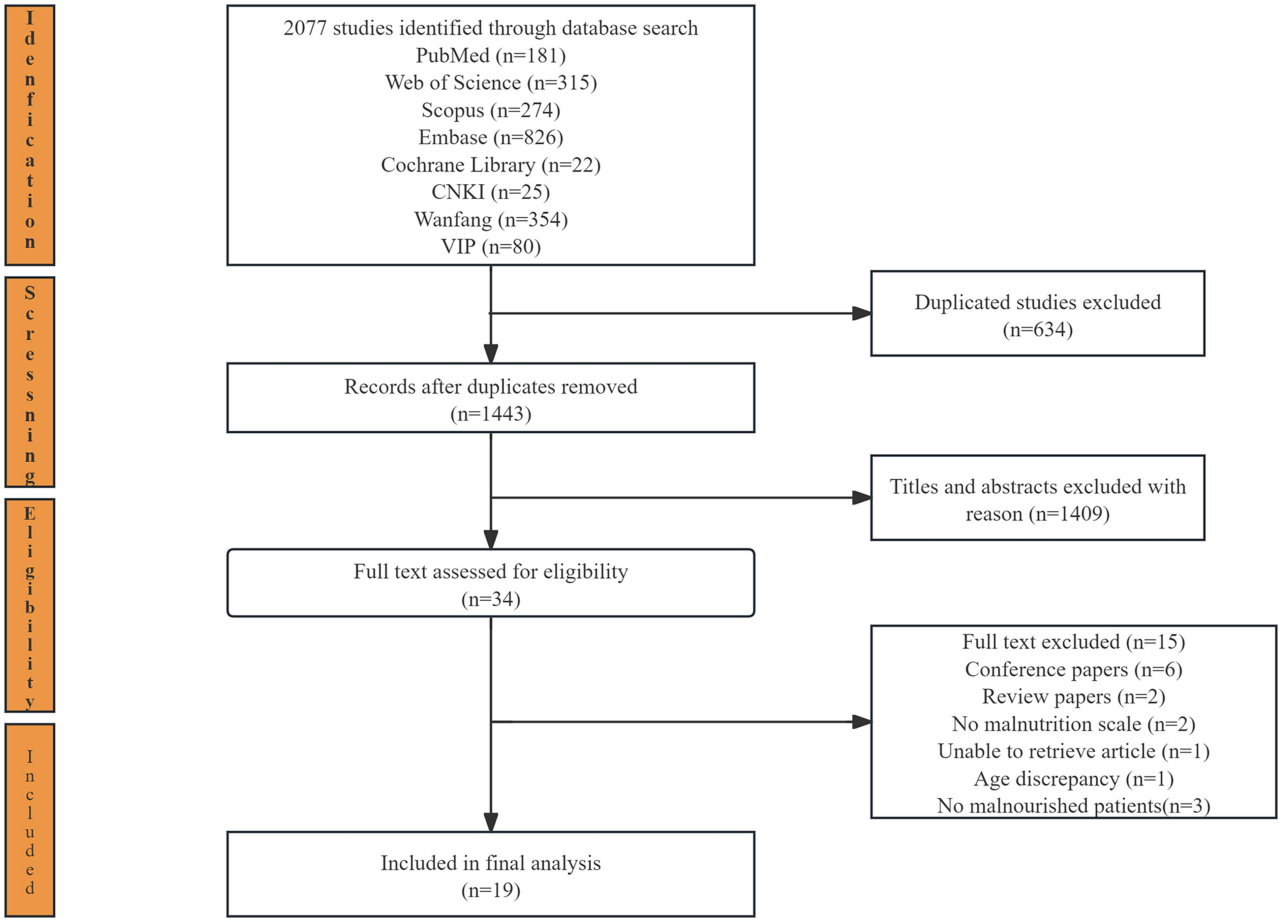
PRISMA flow diagram of search results for included studies.

A total of 2,077 relevant records were identified, of which 634 were duplicates. A total of 1,409 articles were excluded based on the inclusion and exclusion criteria after reading the titles and abstracts. After reviewing the full texts of 34 articles, six conference papers, two reviews, three articles without malnourished patients, two articles without the malnutrition scoring tool used, one article without access to the full text, and one article with an age mismatch were excluded. Ultimately, 19 studies (34–52) (one study without the number of people with malnutrition in CF; and one without the association between CF and malnutrition) were included in this systematic review and meta-analysis. The selection process is illustrated in Figure 1.

### 3.2 Study characteristics

The study characteristics are summarized in Table 1. The 19 studies (34–52) included in this review were all cross-sectional in design, with data collection spanning from 2015 to 2023 and involving 9,987 participants. The sample size ranged from 106 to 4,103 individuals, encompassing most provinces in China. One study (44) had a sample drawn from a nursing home, while others included participants from hospitals and community settings. Eight studies (36, 39, 40, 44, 46, 48, 50, 52) provided no specific age data; however, the inclusion criteria in these studies required participants to be aged 60 years or older. For CF diagnosis, 17 studies (35, 37–52) primarily used the Frailty Phenotype (FP) to assess physical frailty, while only two studies (34, 36) utilized the Frail Scales (FS). Cognitive impairment was assessed using the Mini-Mental State Examination (MMSE) in 12 studies (34, 36, 39–43, 48–52), the Montreal Cognitive Assessment (MoCA) in five studies (37, 44–47), the CDR in one study (35), and the Short Portable Mental Status Questionnaire (SPMSQ) in one study (38). Malnutrition was diagnosed using the Mini Nutritional Assessment-Short Form (MNA-SF) in 14 studies (34–41, 43, 45–49), the full Mini Nutritional Assessment (MNA) in three studies (42, 44, 50), and the Nutritional Risk Screening-2002 (NRS 2002) in two studies (51, 52).

**Table 1:** General characteristics and AHRQ methodological quality of the studies included in the final analysis.

Author (year)	Investigation time	City or province	SS	Mean or median age of the population	Sample size	Women (%)	Cognitive frailty assessment		Definition of malnutrition	Prevalence of malnutrition of CF	Quality score
							Frailty	Cognitive impairment			
Xuedan Yan et al. (2022) (25)	November 2015 to January 2018	Chengdu	H	75.46 ± 7.76	554	212 (38.3)	FS	MMSE	A	36 (40.9)	7
Yingyong Chen et al. a (2022) (26)	August 2020 to February 2021	Guangzhou	C	68.52 ± 4.71	212	127 (59.9)	FP	CDR	A	16 (38.1)	7
Li et al. (2022) (27)	August to December 2020	Hangzhou	C	NONE	252	104 (41.27)	FS	MMSE	B	15 (68.2)	7
Yang et al. (2021) (28)	August to October 2020	Jinzhou	C	72.65 ± 8.95	674	387 (57.4)	FP	MoCA	B	212 (93.8)	7
Ge et al. (2020) (29)	July 2018 to November 2018	Sichuan, Yunnan, Guizhou, Xinjiang	C	67.8 ± 5.9	4,103	2,392 (58.3)	FP	SPMSQ	C	74 (63.8)	7
Zhang et al. (2023) (30)	February to June 2022	Tianjin	H	NONE	217	153 (70.51)	FP	MMSE	A	15 (48.39)	7
Lin et al. (2023) (31)	March to December 2021	Zhejiang	H	NONE	236	112 (47.5)	FP	MMSE	B	36 (43.37)	5
Bai et al. (2019) (32)	September 2021 to August 2022	Yulin, Suzhou	H	71.16 ± 6.2	231	130 (56.28)	FP	MMSE	B	15 (24.59)	7
Jing Yan et al. (2021) (33)	January to October 2019	Nanning	H	75.76 ± 8. 36	106	62 (58.5)	FP	MMSE	E	22 (84. 6)	6
Zhongjun Wang et al. (2023) (34)	January 2022 to April 2023	Haikou	H	71. 57 ± 6. 81	321	149 (46.4)	FP	MMSE	D	34 (40)	6
Ren et al. (2023) (35)	February to May 2023	Hefei	NH	NONE	438	251 (57.3)	FP	MoCA	E	23 (18.1)	7
Yingyong Chen et al. (2022) (36)	August 2020 to March 2021	Guangzhou	C	68	526	289 (54.9)	FP	MoCA	D	60 (43.5)	7
Xiaowei Wang et al. (2023) (37)	January 2022 to January 2023	Shanghai	H	NONE	262	125 (47.71)	FP	MoCA	B	15 (17.6)	7
Liu et al. (2021) (38)	April to December 2019	Urumqi	H	75.3 ± 6.8	395	229 (60)	FP	MoCA	D	43 (52.4)	8
Chengcheng Chen et al. (2023) (39)	May to December 2021	Wenzhou	H	NONE	268	165 (61.6)	FP	MMSE	B	79 (45.1)	6
Wei et al. (2023) (40)	April to October 2022	Beijing	H	71.02 ± 7.93	379	150 (39.6)	FP	MMSE	B	27 (18.6)	6
Yan Wang et al. (2022) (41)	January 2020 to January 2022	Yangzhou	H	NONE	221	NONE	FP	MMSE	E	NONE	6
Jiang et al. (2022) (42)	March 2019 to March 2022	Beijing	H	68.02 ± 6.41	350	182 (52.9)	FP	MMSE	F	18 (58.06)	6
Fan et al. (2021) (43)	June 2020 to February 2021	Beijing	H	NONE	242	74 (30.6)	FP	MMSE	F	31 (26.1)	8

SS: Simple source; H: Hospital; C: Community; NH: Nursing Home; A: MNA-SF (≤ 11 was considered malnutrition); B: MNA-SF (≤7 was considered malnutrition); C: MNA-SF (≤ 12 was considered malnutrition); D: MNA-SF (< 11 was considered malnutrition); E: MNA (< 17 was considered malnutrition); F: NRS-2002; MNA-SF: Mini Nutritional Assessment Short-Form; FP: Frailty Phenotyp; MoCA: the Montreal Cognitive Assessment; NRS2002: the Nutrition Risk screening-2002; FS: FRAIL scale; CDR: Clinical Dementia Rating; PF: physical frailty; MMSE: Mini Mental State Examination; SPMSQ: Short Portable Mental Status Questionnaire; MNA: Mini Nutritional Assessment.

### 3.3 Quality appraisal

Table 1 displays the quality scores of the 19 studies included. All studies were cross-sectional and evaluated using the AHQR scorsing system. The average score was 6.68, with scores ranging from 5 to 8. The majority of studies were “maderate quality,” with scores between 5 to 7, but most scores was 7, while two studies (47, 52) classified as “high quality.” No studies were excluded from our analysis due to low study quality.

All the studies included in research performed well in terms of design description, study rationale, and basic methodology, demonstrating consistency in their underlying framework and theoretical basis. However, most studies exhibit certain shortcomings in controlling bias, describing key variables, and detailing methodological aspects. This suggests that these potential bias factors should be cautiously considered when interpreting the results or conducting a synthesis analysis.

### 3.4 Meta-analysis

#### 3.4.1 CF prevalence in China

**Figure 2:**
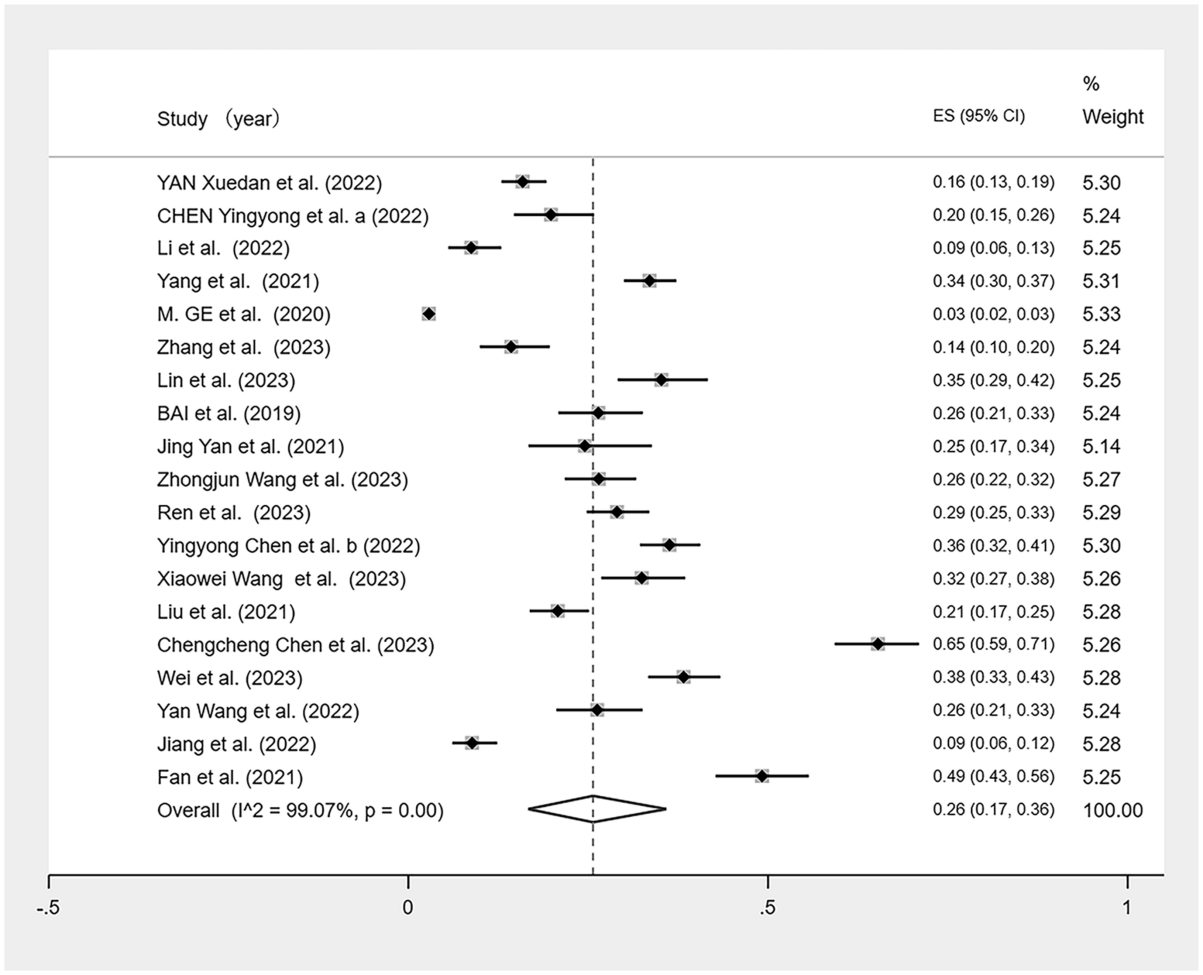
Forest plot of the prevalence of cognitive frailty among older adults in China.

We conducted a meta-analysis of the 19 included studies (Figure 2) using a random-effects model because of the high heterogeneity (I^2^ = 99.07%, *p* < 0.01). The analysis revealed that CF prevalence among older adults aged ≥60 in China is 26% (95% CI: 0.17–0.36, *p* < 0.01).

**Figure 3:**
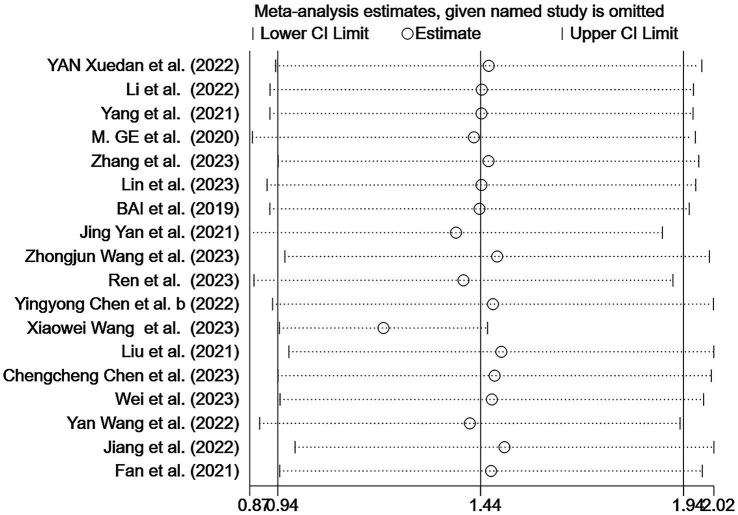
Sensitivity analysis of the prevalence of cognitive frailty among older adults in China.

Sensitivity analyses were conducted on the results; after excluding individual studies, no significant difference exists between the overall combined effect size and the original effect size, indicating that our data were stable (Figure 3). Egger’s and Begg’s tests were used to assess the publication bias. Begg’s test showed no evidence of bias (*p* > 0.05); however, Egger’s test indicated a potential publication bias (*p* < 0.05). Although three different parametric (linear, run, and quadratic) trim-and-fill methods were used to check for publication bias, all methods yielded an effect size of 0.269 (95% CI: 0.207–0.331) with no additional studies filled, suggesting no significant publication bias and confirming the robustness of the study.

#### 3.4.2 Malnutrition prevalence in patients with CF in China

**Figure 4:**
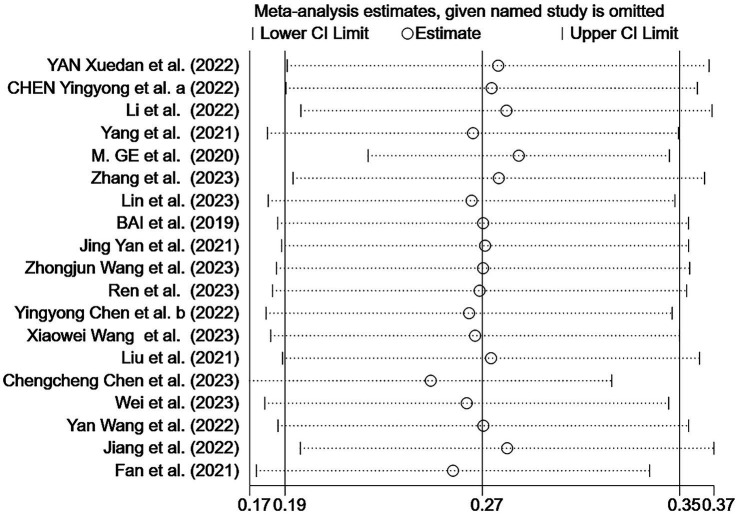
Forest plot of the malnutrition prevalence in older Chinese adults with cognitive frailty.

Of the 19 studies, one (50) lacked data on the prevalence of malnutrition among patients with CF. A meta-analysis was conducted on the remaining 18 studies (Figure 4). Given the high heterogeneity observed (I^2^ = 96.7%, *p* < 0.01), a random effects model was used. The meta-analysis showed malnutrition prevalence of 45% (95% CI: 0.32–0.58, *p* < 0.01).

**Figure 5:**
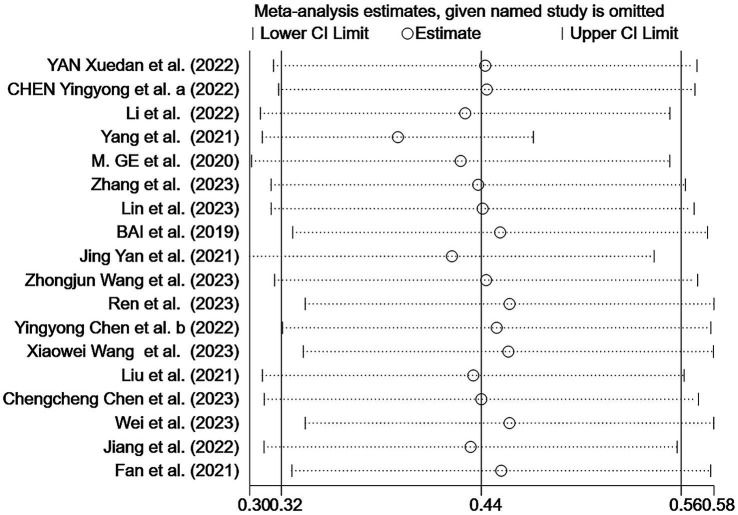
Sensitivity analysis of the malnutrition prevalence among older Chinese adults with cognitive frailty.

The sensitivity analysis indicated the stability of our findings (Figure 5). Begg’s test showed no bias (*p* > 0.05), but Egger’s test indicated potential publication bias (*p* < 0.05). Using three different non-parametric trim-and-fill methods (linear, run, and quadratic), the effect size remained constant at 0.438 (95% CI: 0.332–0.544) without including any additional studies, suggesting that the study’s results were robust.

#### 3.4.3 Association between CF and malnutrition in China

**Figure 6:**
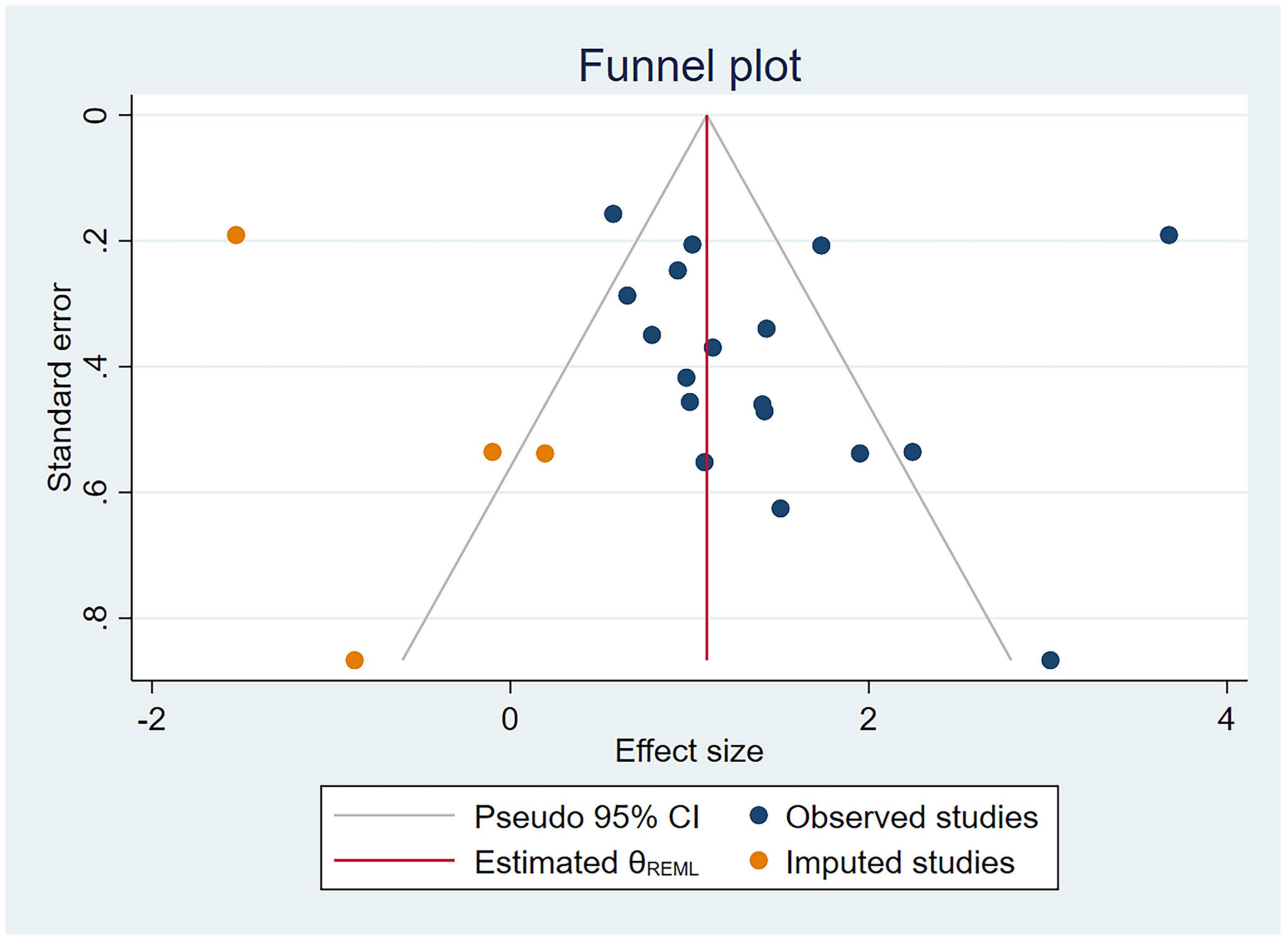
Forest plot of the association between cognitive frailty and malnutrition in older Chinese adults.

**Figure 7:**
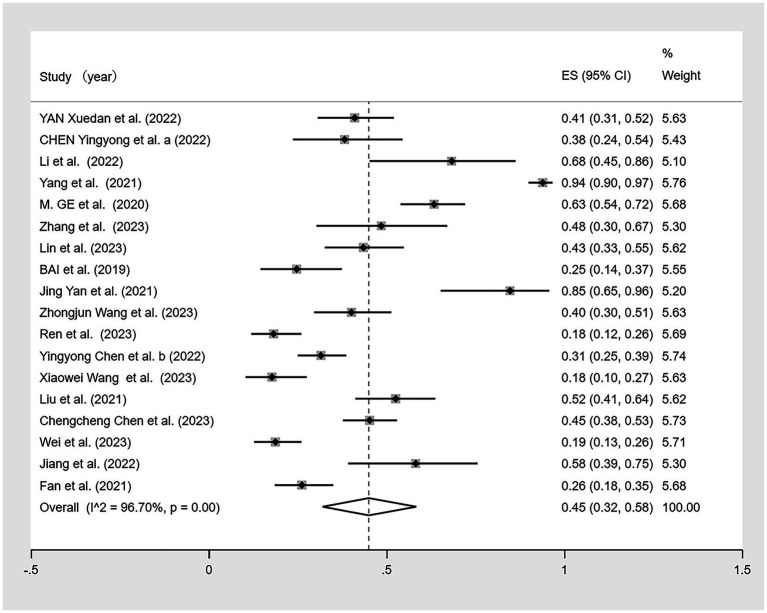
Sensitivity analysis of the association between cognitive frailty and malnutrition in older Chinese adults.

**Figure 8:**
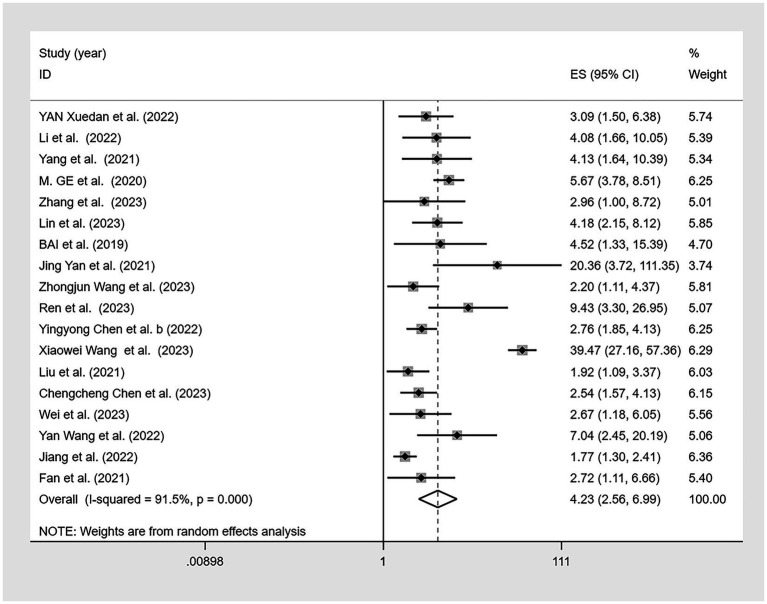
Trim-and-fill funnel plot for the association between cognitive frailty and malnutrition in older Chinese adults.

One study (35) lacked an odds ratio (OR) for the association between CF and malnutrition. Therefore, a meta-analysis was performed on the remaining 18 studies (Figure 6), revealing a significant association between CF and malnutrition (OR = 4.23, 95% CI: 2.56–6.99, *p* < 0.001). Although there was high heterogeneity (I^2^ = 91.5%, *p* < 0.001), the sensitivity analysis indicated good stability of the results (Figure 7). Both Egger’s and Begg’s tests demonstrated bias (*p* < 0.05), suggesting a potential publication bias. To address this, the trim-and-fill method was applied to detect and correct for publication bias (Figure 8). With this adjustment, and after adding four additional studies, the funnel plot became symmetric, slightly reducing the effect size to 1.097 (95% CI: 1.034–1.826). Following logarithmic transformation, the OR was 3.00 (95% CI: 1.87–4.80).

#### 3.4.4 Subgroup analyses

##### 3.4.4.1 Malnutrition prevalence in patients with CF in China

To identify heterogeneity sources, we conducted several subgroup analyses (Table 2). The prevalence of malnutrition in CF diagnosed by MMSE and MoCA was 43% (95% CI: 0.33–0.54) and 44% (95% CI: 0.12–0.79), respectively. Others (CDR and SPMSQ) accounted for 57% (95% CI: 0.49–0.64). In 18 studies based on hospital and community samples, the prevalence was 38% (95% CI: 0.29–0.47) in hospitals and 61% (95% CI: 0.29–0.89) in the community. Studies were grouped according to different diagnostic tools and MNA-SF definition. Malnutrition prevalence diagnosed with MNA-SF (≤7 scores) was 45% (95% CI: 0.35–0.55), and the prevalence diagnosed with MNA-SF (1–11 scores) was 45% (95% CI: 0.18–0.73), MNA diagnosis was 28% (95% CI: 0.21–0.36), and NRS 2002 diagnosis was 32% (95% CI: 0.25–0.40).

**Table 2:** Subgroup analyses about prevalence of malnutrition in CF patients.

Subgroups	Studies (*n*)	Prevalence (%)	95% CI	Heterogeneity		Model
				I^2^(%)	*P*	
Diagnosis of cognitive impairment						
MMSE	11	43	0.33–0.54	88.13	<0.01	Random
MoCA	5	44	0.12–0.79	98.95	<0.01	Random
Others	2	57	0.49–0.64	0	/	Random
Diagnosis of frailty						
FP	16	44	0.30–0.58	97.06	<0.01	Random
FS	2	46	0.37–0.56	0	/	Random
Simple source						
Hospital	13	38	0.29–0.47	89.85	<0.01	Random
Community	5	61	0.29–0.89	98.27	<0.01	Random
Scales and definition of malnutrition						
MNA-SF (≤7 scores)	7	45	0.35–0.55	82.5	<0.01	Random
MNA-SF (1–11 scores)	7	45	0.18–0.73	98.43	<0.01	Random
MNA	2	28	0.21–0.36	0	/	Random
NRS2002	2	32	0.25–0.40	0	/	Random

MMSE: Mini Mental State Examination; MoCA: the Montreal Cognitive Assessment; FP: Frailty Phenotyp; FS: FRAIL scale; MNA-SF: Mini Nutritional Assessment Short-Form; MNA: Mini Nutritional Assessment; NRS2002: the Nutrition Risk screening-2002.

##### 3.4.4.2 Association between CF and malnutrition

Subgroup analysis was conducted for the association between malnutrition and CF. We categorized data based on cognitive impairment measurement scales, tools assessing physical frailty, sample sources, and malnutrition diagnostic tools. Owing to the varying diagnostic criteria within the MNA-SF, we further subdivided the MNA-SF malnutrition groups for a more detailed subgroup analysis (Table 3). Using the MMSE to diagnose CF, the pooled OR was 3.02 (95% CI: 2.27–4.02), while the MoCA yielded an OR of 6.07 (95% CI: 1.51–24.31). Of 15 studies, the FP diagnostic tool for malnutrition associated with CF indicated an OR of 4.33 (95% CI: 2.49–7.52), and the FS an OR of 3.45 (95% CI: 1.96–6.07). Regarding sample sources, the association between CF and malnutrition in hospital settings had an OR of 4.09 (95% CI: 2.02–8.27) and 4.00 (95% CI: 2.64–6.06; in community settings). For malnutrition diagnostic tools, the OR for CF with malnutrition using the MNA-SF scores ≤7 was 5.15 (95% CI: 1.78–14.91) and 3.00 (95% CI: 2.06–4.37) for scores 0–11. The comprehensive MNA showed an OR of 9.45 (95% CI: 4.78–18.67). Using the NRS 2002 for diagnosing malnutrition in these patients resulted in an OR of 1.86 (95% CI: 1.39–2.48).

**Table 3:** Subgroup analyses about association between CF and malnutrition.

Subgroups	Studies (*n*)	OR (95%CI)	Heterogeneity		Model
			I^2^(%)	*P*	
Diagnosis of cognitive impairment					
MMSE	12	3.02*** (2.27–4.02)	42.6	0.058	Random
MoCA	5	6.07* (1.51–24.31)	96.8	<0.001	Random
Diagnosis of frailty					
FP	15	4.33*** (2.49–7.52)	92.4	<0.001	Random
FS	2	3.45*** (1.96–6.07)	0	0.639	Random
The different of definition and scales of malnutrition					
MNA-SF ≤7 scores	7	5.15** (1.78–14.91)	94.4	<0.001	Random
MNA-SF 0–11 scores	6	3.00*** (2.06–4.37)	59.9	<0.001	Random
MNA	3	9.45*** (4.78–18.67)	0	0.581	Random
NRS2002	2	1.86*** (1.39–2.48)	0	0.375	Random
Simple source					
Hospital	13	4.09*** (2.02–8.27)	93.7	<0.001	Random
Community	4	4*** (2.64–6.06)	50.6	0.108	Random

MMSE: Mini Mental State Examination; MoCA: the Montreal Cognitive Assessment; FP: Frailty Phenotyp; FS: FRAIL scale; MNA-SF: Mini Nutritional Assessment Short-Form; MNA: Mini Nutritional Assessment; NRS2002: the Nutrition Risk screening-2002; *: *P* < 0.05; **: *P* < 0.01; ***: *P* < 0.001.

## 4 Discussion

To our knowledge, this is the first systematic review and meta-analysis on the prevalence and association of malnutrition with CF in China. Our findings indicate that the current CF prevalence in older Chinese adults is 26%, with 45% of the population facing malnutrition. Notably, a significant association occurred between CF and malnutrition (OR = 4.23), adjusted to 3.00 after applying trim-and-fill analysis for bias correction.

We conducted sensitivity analyses using the Egger’s and Begg’s tests. If biases were identified, then the results were adjusted using the trim-and-fill method to ensure reliability. CF prevalence (26%) in our study differed significantly from the 15% reported by Liu et al. (10), which may be due to differences in the diagnostic criteria for CF, study time, sample size, age, and sample source. Compared with Liu et al., our study is closer to the present time, covering data from 2015 to 2023. Although Liu et al. included a larger sample size and regions including Hong Kong and Taiwan, our data may more accurately reflect the recent prevalence in mainland China. Moreover, as age increases, the decline in bodily functions, such as reduced gastrointestinal function, can impair digestion and absorption, thereby increasing the risk of malnutrition (18). Additionally, CF is diagnosed based on the presence of both physical frailty and cognitive impairment. Physical frailty typically results in reduced muscle mass, which restricts the activity levels of older adults and may make adequate food intake more challenging, which in turn exacerbates the risk of malnutrition (18).

Our study revealed that malnutrition prevalence in patients with CF in China is 45%, which remains robust after validation using a sensitivity analysis and the trim-and-fill method. According to Song et al. (17), malnutrition prevalence among community-dwelling older adults in China is 41.8%. However, since 2017, a significant decline occurred in malnutrition rates among older adults, from 66.6% between 2011 and 2016 to 29.6% between 2017 and 2021. This decline appears to be closely linked to a series of health strategies and policy initiatives implemented in recent years.

Several factors likely contributed to this improvement. First, the “Health China 2030” goals was established and the National Nutrition Plan of Action (2017–2030) was issued, which outlines multi-sectoral transdisciplinary measures to improve population nutrition status (53). Second, the dietary patterns among the Chinese population have changed from lacking animal-source foods, dairy products, and fruits to excessive intake of meat (especially pork) and fats, which reflects an overall improvement in the food supply and economy (54). Third, in 2023 the national standard “Basic Specification for At-home Care Services for older adults” (GB/T 43153-2023) was issued, mandating personalized dietary assistance and regular health assessments to enable the timely identification and correction of nutritional problems (55). Additionally, from an economic perspective, increased pension benefits and targeted poverty alleviation policies have provided older adults with greater financial resources, thereby enhancing their ability to invest in higher-quality food (56).

Despite these positive trends, most of the studies included in this review were published after 2020; however, the malnutrition prevalence in these studies remained significantly higher than the reported prevalence of 29.6%. Malnutrition not only adversely affects the overall health status of older adults but also significantly increases the rates of hospital readmissions and mortality (57). However, the prevalence among older patients is markedly higher than that in the normal older population. Therefore, for older patients with CF, it is imperative that medical and nursing institutions strengthen nutritional assessments and interventions.

In the subgroup analysis, we considered the impact of using different assessment scales. Diagnoses of physical frailty within the CF were categorized using the FP and FS; cognitive impairment was grouped based on the MMSE, MoCA, and others (CDR + SPMSQ). We found that malnutrition prevalence was similar across the physical frailty diagnoses of FP and FS and cognitive impairments diagnosed using the MMSE and MoCA, indicating that malnutrition is commonly observed in CF, with prevalence rates ranging from 43 to 46%. This similarity suggests that these scales are reliable for diagnosing CF and highlights the significant presence of malnutrition in this condition.

Subgroup analysis based on the sample source showed that CF prevalence in hospital settings was lower than that in community settings. However, the prevalence among hospitalized patients with CF (38%) exceeds the range of 19.3–27.8% observed in the general hospitalized population (58). Similarly, the prevalence in the community was significantly higher than the 41.2% prevalence reported in the general community in China (17), further underscoring the close association between CF and malnutrition. Although our results showed similar associations between CF and malnutrition in both the community and hospital settings, the marked differences in prevalence rates raise a critical question: what causes such a large disparity? These differences may arise from various factors, including variations in CF definitions, assessment methods, and characteristics of survey participants. Additionally, environmental factors such as health status and living conditions may influence the prevalence of malnutrition in community and hospital settings (59). In hospital settings, standardized malnutrition screening tools are routinely used to assess patients, thereby enhancing the early identification of malnutrition (60). In contrast, in community settings, the lack of timely patient assessments and limited use of standardized screening tools often results in malnutrition being overlooked, which in turn leads to a higher reported prevalence of malnutrition (61). Moreover, community healthcare institutions frequently face shortages of personnel, funding, and resources, making it challenging to conduct large-scale, systematic nutritional screening and monitoring; consequently, nutritional interventions are implemented less promptly and effectively (61). Conversely, hospitals benefit from centralized management and adequate resource allocation, enabling them to promptly adjust nutritional support—whether via enteral or parenteral nutrition—as patients’ conditions change, which further explains why significant differences in nutritional status may be observed between community and hospital contexts (62). Given these factors, understanding the nutritional status of patients with CF is paramount. Prioritizing the management of malnutrition among community-dwelling individuals with CF is essential to develop effective intervention strategies and improve their nutritional health.

This study also discusses the relationship between malnutrition and CF. Considering that various malnutrition diagnostic tools were used in this study, the diagnostic criteria for malnutrition may vary across different tools. Malnutrition was differentiated using the MNA-SF scale, as defined by scores of 0–7 and 0–11 (excluding studies that only considered 0–7). Malnutrition prevalence was consistent across both MNA-SF groups, possibly due to the similar range of scores among patients with CF. This finding supports the effectiveness of the MNA-SF as a tool for assessing malnutrition in patients with CF. Other tools assessed malnutrition prevalence at rates lower than those found using the MNA-SF, possibly because of the smaller sample sizes in these studies, which could have influenced the outcomes. However, the MNA and NRS 2002 are also reliable tools for diagnosing malnutrition and have been validated in numerous studies (63, 64). The observed discrepancies may result from differences in environmental factors, underlying health conditions, and specificity and sensitivity of the screening tools used (65, 66).

We showed that the association between CF and malnutrition (OR = 3.00) was similar to that of other diseases, such as diabetes, cardiovascular diseases, and depression, and was attributable to several mechanisms (67, 68). First, malnutrition can directly cause physical frailty and cognitive impairment (69, 70). Second, an interplay exists between physical frailty and cognitive impairment, where the deterioration of one can worsen the symptoms of the other (71). CF is defined as the simultaneous presence of physical frailty, and cognitive impairment was defined as CF (8). Therefore, CF and malnutrition are more closely related than other diseases and are risk factors for each other, jointly affecting the health status of patients.

In our subgroup analysis, we observed significant differences in the association between malnutrition and CF diagnosed using the MMSE and MoCA, due to the higher heterogeneity associated with the MoCA, influenced by the study design, sample characteristics, and assessment criteria. Furthermore, the MoCA is more sensitive to mild cognitive impairment compared with the MMSE, indirectly enhancing the detection of associations with malnutrition (72).

In our subgroup analysis, we observed significant differences among the three scales, attributable to the different CF diagnostic methods, different sources of the sample population, and different sensitivities and characteristics of each scale (66). For example, we noted a high level of heterogeneity in the results between the two MNA-SF groups, stemming from variations in CF diagnostic methods and the populations studied. Additionally, the MNA-SF results indicated a closer relationship between severe malnutrition and CF. Lower results might be influenced by the study of Yan et al. (42), which included both malnutrition and the risk of malnutrition. Compared with other scales, the NRS 2002, owing to its higher specificity and positive likelihood ratio, more accurately diagnose malnutrition, potentially reflecting a more accurate association between CF and malnutrition (66). Regardless of the scale, a significant association was observed between CF and malnutrition. Therefore, the diagnosis and treatment of malnutrition in patients with CF should be emphasized.

One of the strengths of our study approach was that we comprehensively searched for both Chinese and English studies, which mitigated the risk of selection bias. Sensitivity analysis and subgroup analysis were conducted on the research results to explore the possible causes of heterogeneity. Egger’s and Begg’s tests were performed to verify the accuracy of the results. Additionally, the trim-and-fill method was used to address potential bias, thereby bolstering the rigor of our findings.

Despite our promising results, our research had the following limitations. The high heterogeneity in our studies may be related to differences in population culture and characteristics due to the included study samples, research methods, and regional populations. Current studies using a cross-sectional method could not accurately prove the causal relationship between CF and malnutrition in the included studies. Furthermore, the lack of standardized diagnostic criteria for malnutrition and CF across the included studies may have resulted in slight discrepancies in the findings.

Future research should prioritize prospective studies to better determine the causal relationships between CF and malnutrition. Standardizing the assessment tools for CF and malnutrition is essential to facilitate a comprehensive comparison of the results across studies. For older adults with CF in China, researchers, clinicians, nurses, and policymakers should focus on malnutrition, particularly in community settings, implementing preventive and interventional measures to reduce malnutrition incidence associated with CF.

## 5 Conclusion

This study investigated the prevalence of CF and malnutrition among older adults in China and their association. The prevalence of CF was found to be 26%, with 45% of individuals with CF experiencing malnutrition. A significant correlation between CF and malnutrition was observed, with an adjusted OR adjusted of 3.00. The study also highlighted a higher prevalence of malnutrition among community-dwelling individuals with CF compared with those in hospital settings. These findings underscore the importance of preventive and intervention strategies to address CF and malnutrition in the Chinese older population.

## Data Availability

The original contributions presented in the study are included in the article/Supplementary material, further inquiries can be directed to the corresponding author.
