# Supplementary material for: Association of malnutrition with cognitive frailty in China: a systematic review and meta-analysis
Source: Front Public Health. 2025 Apr 14;13:1567372. doi: 10.3389/fpubh.2025.1567372 (PMC12034679; doi:10.3389/fpubh.2025.1567372)
Supplement: Supplementary file 1 [file Data_Sheet_1.docx]

**Supplementary File**

Association of malnutrition with cognitive frailty in China: A systematic review and meta-analysis

**Contents**

**Supplementary Table 1 PRISMA checklist for this systematic review and meta-analysis**

**Supplementary Table 2 Detailed search strategy in all databases**

**Supplementary Table 3 The (bias) quality assessment of included studies (Agency for Healthcare Research and Quality [AHRQ])**

**Supplementary Figure 1 Funnel Plot of Cognitive Frailty Prevalence and Trim-and-Fill Methods: A Funnel Plot, B Trim-and-Fill Run Method, C Trim-and-Fill Quadratic Method, D Trim-and-Fill Linear Method**

**Supplementary Figure 2 Funnel Plot of Malnutrition Prevalence in Cognitive Frailty and Trim-and-Fill Methods: A Funnel Plot, B Trim-and-Fill Run Method, C Trim-and-Fill Quadratic Method, D Trim-and-Fill Linear Method**

**Supplementary Figure 3 The prevalence of Malnutrition in Cognitive Frailty assessed using different cognitive impairment diagnostic tools**

**Supplementary Figure 4 The prevalence of malnutrition in Cognitive Frailty assessed using different frailty diagnostic tools**

**Supplementary Figure 5 The prevalence of Malnutrition with Cognitive Frailty in different simple source**

**Supplementary Figure 6 The prevalence of Malnutrition diagnosed using different diagnostic tools among Cognitive Frailty**

**Supplementary Figure 7 The association between malnutrition and Cognitive Frailty diagnosed using different cognitive function assessment tools**

**Supplementary Figure 8 The association between Malnutrition and Cognitive Frailty diagnosed using different frailty assessment tools**

**Supplementary Figure 9 The association between Malnutrition and Cognitive Frailty diagnosed using different Malnutrition assessment tools**

**Supplementary Figure 10 The association between Malnutrition and Cognitive Frailty in different Simple source**

**Supplementary Figure 11 Funnel Plot of the Association Between Cognitive Frailty and Malnutrition**

# Supplementary Tables

## Supplementary Table 1 PRISMA checklist for this systematic review and meta-analysis

| **Section and Topic** | **Item #** | **Checklist item** | **Location where item is reported** |
| --- | --- | --- | --- |
| **TITLE** | | |  |
| Title | 1 | Identify the report as a systematic review. | P.1 |
| **ABSTRACT** | | |  |
| Abstract | 2 | See the PRISMA 2020 for Abstracts checklist. | P.2 |
| **INTRODUCTION** | | |  |
| Rationale | 3 | Describe the rationale for the review in the context of existing knowledge. | P.3-4 |
| Objectives | 4 | Provide an explicit statement of the objective(s) or question(s) the review addresses. | P.4-5 |
| **METHODS** | | |  |
| Eligibility criteria | 5 | Specify the inclusion and exclusion criteria for the review and how studies were grouped for the syntheses. | P.5-6 |
| Information sources | 6 | Specify all databases, registers, websites, organisations, reference lists and other sources searched or consulted to identify studies. Specify the date when each source was last searched or consulted. | P.5 |
| Search strategy | 7 | Present the full search strategies for all databases, registers and websites, including any filters and limits used. | P.5 |
| Selection process | 8 | Specify the methods used to decide whether a study met the inclusion criteria of the review, including how many reviewers screened each record and each report retrieved, whether they worked independently, and if applicable, details of automation tools used in the process. | P.6 |
| Data collection process | 9 | Specify the methods used to collect data from reports, including how many reviewers collected data from each report, whether they worked independently, any processes for obtaining or confirming data from study investigators, and if applicable, details of automation tools used in the process. | P.6-7 |
| Data items | 10a | List and define all outcomes for which data were sought. Specify whether all results that were compatible with each outcome domain in each study were sought (e.g. for all measures, time points, analyses), and if not, the methods used to decide which results to collect. | P.6-7 |
|  | 10b | List and define all other variables for which data were sought (e.g. participant and intervention characteristics, funding sources). Describe any assumptions made about any missing or unclear information. | P.6-7 |
| Study risk of bias assessment | 11 | Specify the methods used to assess risk of bias in the included studies, including details of the tool(s) used, how many reviewers assessed each study and whether they worked independently, and if applicable, details of automation tools used in the process. | P.7-8 |
| Effect measures | 12 | Specify for each outcome the effect measure(s) (e.g. risk ratio, mean difference) used in the synthesis or presentation of results. | P.7-8 |
| Synthesis methods | 13a | Describe the processes used to decide which studies were eligible for each synthesis (e.g. tabulating the study intervention characteristics and comparing against the planned groups for each synthesis (item #5)). | - |
|  | 13b | Describe any methods required to prepare the data for presentation or synthesis, such as handling of missing summary statistics, or data conversions. | - |
|  | 13c | Describe any methods used to tabulate or visually display results of individual studies and syntheses. | - |
|  | 13d | Describe any methods used to synthesize results and provide a rationale for the choice(s). If meta-analysis was performed, describe the model(s), method(s) to identify the presence and extent of statistical heterogeneity, and software package(s) used. | P.7-8 |
|  | 13e | Describe any methods used to explore possible causes of heterogeneity among study results (e.g. subgroup analysis, meta-regression). | P.7-8 |
|  | 13f | Describe any sensitivity analyses conducted to assess robustness of the synthesized results. | P.8 |
| Reporting bias assessment | 14 | Describe any methods used to assess risk of bias due to missing results in a synthesis (arising from reporting biases). | - |
| Certainty assessment | 15 | Describe any methods used to assess certainty (or confidence) in the body of evidence for an outcome. | P.8 |
| **RESULTS** | | |  |
| Study selection | 16a | Describe the results of the search and selection process, from the number of records identified in the search to the number of studies included in the review, ideally using a flow diagram. | P.8 and  Figure. 1 |
|  | 16b | Cite studies that might appear to meet the inclusion criteria, but which were excluded, and explain why they were excluded. | P.8 |
| Study characteristics | 17 | Cite each included study and present its characteristics. | P.8-9, and Table 1 |
| Risk of bias in studies | 18 | Present assessments of risk of bias for each included study. | P.9, Table 1 and Appendix 2 |
| Results of individual studies | 19 | For all outcomes, present, for each study: (a) summary statistics for each group (where appropriate) and (b) an effect estimate and its precision (e.g. confidence/credible interval), ideally using structured tables or plots. | Table 1 |
| Results of syntheses | 20a | For each synthesis, briefly summarise the characteristics and risk of bias among contributing studies. | Appendix 2 |
|  | 20b | Present results of all statistical syntheses conducted. If meta-analysis was done, present for each the summary estimate and its precision (e.g. confidence/credible interval) and measures of statistical heterogeneity. If comparing groups, describe the direction of the effect. | Figure. 2 |
|  | 20c | Present results of all investigations of possible causes of heterogeneity among study results. | P.10-11 |
|  | 20d | Present results of all sensitivity analyses conducted to assess the robustness of the synthesized results. | P10-11 |
| Reporting biases | 21 | Present assessments of risk of bias due to missing results (arising from reporting biases) for each synthesis assessed. | Not applicable |
| Certainty of evidence | 22 | Present assessments of certainty (or confidence) in the body of evidence for each outcome assessed. | Not applicable |
| **DISCUSSION** | | |  |
| Discussion | 23a | Provide a general interpretation of the results in the context of other evidence. | P.13 |
|  | 23b | Discuss any limitations of the evidence included in the review. | P.18 |
|  | 23c | Discuss any limitations of the review processes used. | P.18 |
|  | 23d | Discuss implications of the results for practice, policy, and future research. | P.18 |
| **OTHER INFORMATION** | | |  |
| Registration and protocol | 24a | Provide registration information for the review, including register name and registration number, or state that the review was not registered. | P.5 |
|  | 24b | Indicate where the review protocol can be accessed, or state that a protocol was not prepared. | Not applicable |
|  | 24c | Describe and explain any amendments to information provided at registration or in the protocol. | Not applicable |
| Support | 25 | Describe sources of financial or non-financial support for the review, and the role of the funders or sponsors in the review. | P.20 |
| Competing interests | 26 | Declare any competing interests of review authors. | P.20 |
| Availability of data, code and other materials | 27 | Report which of the following are publicly available and where they can be found: template data collection forms; data extracted from included studies; data used for all analyses; analytic code; any other materials used in the review. | P.20 |

## Supplementary Table 2 Detailed search strategy in all databases

| Database | Search terms | Number |
| --- | --- | --- |
| PubMed | ((((((Malnutrition[MeSH Terms]) OR (Malnutrition[Title/Abstract])) OR (Nutritional Deficiency[Title/Abstract])) OR (Nutritional Deficiencies[Title/Abstract])) OR (Undernutrition[Title/Abstract])) OR (Malnourishment[Title/Abstract])) OR (Malnourishments[Title/Abstract]) | 180377 |
|  | (((Frailty[MeSH Terms]) OR (frail*[Title/Abstract])) OR (Debilit*[Title/Abstract])) OR (Debilities[Title/Abstract]) | 75464 |
|  | (Cognitive decline[Title/Abstract]) OR (Cognitive impairment[Title/Abstract]) | 113728 |
|  | ((((Frailty[MeSH Terms]) OR (frail*[Title/Abstract])) OR (Debilit*[Title/Abstract])) OR (Debilities[Title/Abstract])) AND ((Cognitive decline[Title/Abstract]) OR (Cognitive impairment[Title/Abstract])) | 3045 |
|  | Cognitive frailty[Title/Abstract] | 386 |
|  | (cognitive frailty[Title/Abstract]) OR (((((Frailty[MeSH Terms]) OR (frail*[Title/Abstract])) OR (Debilit*[Title/Abstract])) OR (Debilities[Title/Abstract])) AND ((Cognitive decline[Title/Abstract]) OR (Cognitive impairment[Title/Abstract]))) | 3180 |
|  | (((((((Malnutrition[MeSH Terms]) OR (Malnutrition[Title/Abstract])) OR (Nutritional Deficiency[Title/Abstract])) OR (Nutritional Deficiencies[Title/Abstract])) OR (Undernutrition[Title/Abstract])) OR (Malnourishment[Title/Abstract])) OR (Malnourishments[Title/Abstract])) AND ((((((Frailty[MeSH Terms]) OR (frail*[Title/Abstract])) OR (Debilit*[Title/Abstract])) OR (Debilities[Title/Abstract])) AND ((Cognitive decline[Title/Abstract]) OR (Cognitive impairment[Title/Abstract]))) OR (Cognitive frailty[Title/Abstract])) | 181 |
| Web of science | 1#：(((((AB=(Malnutrition)) OR AB=(Nutritional Deficiency)) OR AB=(Nutritional Deficiencies)) OR AB=(Undernutrition)) OR AB=(Malnourishment)) OR AB=(Malnourishments) and Preprint Citation Index | 87707 |
|  | 2#：(((AB=(Frailty)) OR AB=(frail*)) OR AB=(Debilit*)) OR AB=(Debilities) and Preprint Citation Index | 88014 |
|  | 3#：(AB=(Cognitive decline)) OR AB=(Cognitive impairment) | 184994 |
|  | 4# ：#2 AND #3 | 4240 |
|  | 5#：(AB=(Cognitive frailty)) OR TI=(Cognitive frailty) | 4218 |
|  | 6# ：#5 OR #4 | 5950 |
|  | #1 AND #6 | 315 |
| Scopus | 1#：(TITLE-ABS-KEY(malnutrition) OR TITLE-ABS-KEY ("nutritional deficiency") OR TITLE-ABS-KEY ("nutritional deficiencies" ) OR TITLE-ABS-KEY ( undernutrition ) OR TITLE-ABS-KEY ( malnourishment ) OR TITLE-ABS-KEY ( malnourishments ) ) | 143882 |
|  | 2#：(TITLE-ABS-KEY (frailty) OR TITLE-ABS-KEY (frail*) OR TITLE-ABS-KEY (debilit*) OR TITLE-ABS-KEY (debilities) ) | 104417 |
|  | 3#：( TITLE-ABS-KEY ( "cognitive decline" ) OR TITLE-ABS-KEY ( "cognitive impairment" ) ) | [150,949](https://www-scopus-com-s-a550.bj2.80589.org/search/history/results.uri?origin=searchhistory&shid=4) |
|  | 4#：2# AND 3# | 4086 |
|  | 5#：TITLE-ABS-KEY ( "cognitive frailty" ) | 444 |
|  | 6#：5# OR 4# | 4250 |
|  | 7#：6# AND 1# | 274 |
| Embase | 1#:'frailty'/exp OR 'frail*':ta,ab,kw OR 'debilit*':ta,ab,kw | 114119 |
|  | 2#:'malnutrition'/exp OR 'malnutrition' OR 'deficient nutrition':ta,ab,kw OR 'malnourishment':ta,ab,kw OR 'severe acute malnutrition':ta,ab,kw OR 'underfeeding':ta,ab,kw OR 'undernourishment':ta,ab,kw OR 'undernutrition':ta,ab,kw OR 'malnutrition':ta,ab,kw | 241694 |
|  | 3#:'cognitive decline'/exp OR 'cognitive decline' OR 'cognitive impairment'/exp OR 'cognitive impairment' | 672882 |
|  | 4#:1# AND 3# | 11446 |
|  | 5#:#4 AND #7 OR 'cognitive frailty':ta,ab,kw | 11526 |
|  | 6#:2# AND 5# | 826 |
| Cochrane library | 1#:（“malnutrition”）:ab;ti,kw | 6432 |
|  | 2#：("Nutritional Deficiency"):ti,ab,kw OR ("Nutritional Deficiencies"):ti,ab,kw OR ("Undernutrition"):ti,ab,kw OR ("Malnourishments"):ti,ab,kw OR ("Malnourishment"):ti,ab,kw | 1400 |
|  | 3#：("frailty"):ti,ab,kw OR ("frailties"):ti,ab,kw OR ("Debility"):ti,ab,kw OR ("Debilities"):ti,ab,kw OR ("Debility"):ti,ab,kw | 3726 |
|  | 4#：("cognitive decline"):ti,ab,kw OR ("cognitive impairment"):ti,ab,kw | 15427 |
|  | 5#：#3 AND #4 | 309 |
|  | 6#：#5 OR ("cognitive frailty"):ti,ab,kw | 349 |
|  | 7#：#1 OR #2 | 7276 |
|  | 8#：#7 AND #6 | 22 |
| CNKI | (（TKA=（‘虚弱’+‘衰弱’) AND TKA=(‘认知障碍’+‘认知功能下降’）) OR TKA='认知衰弱') AND TKA=营养不良' | 25 |
| Wangfang | （（（题名或关键词:(认知下降) or 题名或关键词:(认知功能障碍) ） ）and （题名或关键词:(虚弱) or 题名或关键词:(衰弱) ）） or 题名或关键词:(认知衰弱) ）and 题名或关键词:(营养不良) | 354 |
| VIP | ((R=(虚弱 OR 衰弱) AND R=(认知下降 OR 认知障碍)) OR R=认知衰弱) AND R=营养不良 | 80 |

## Supplementary Table 3 The (bias) quality assessment of included studies (Agency for Healthcare Research and Quality [AHRQ])

| First author/year | Q1 | Q2 | Q3 | Q4 | Q5 | Q6 | Q7 | Q8 | Q9 | Q10 | Q11 | Total Points |
| --- | --- | --- | --- | --- | --- | --- | --- | --- | --- | --- | --- | --- |
| Xuedan Yan et al (2022) | 1 | 1 | 1 | 1 | 0 | 0 | 1 | 1 | 0 | 1 | 0 | 7 |
| Yingyong Chen et al a (2022) | 1 | 1 | 1 | 1 | 0 | 1 | 1 | 0 | 0 | 1 | 0 | 7 |
| Li et al. (2022) | 1 | 1 | 1 | 1 | 0 | 0 | 1 | 1 | 0 | 1 | 0 | 7 |
| Yang et al. (2021) | 1 | 1 | 1 | 1 | 0 | 1 | 1 | 0 | 0 | 1 | 0 | 7 |
| Ge et al. (2020) | 1 | 1 | 1 | 1 | 0 | 0 | 1 | 1 | 0 | 1 | 0 | 7 |
| Zhang et al. (2023) | 1 | 1 | 1 | 1 | 0 | 0 | 1 | 1 | 0 | 1 | 0 | 7 |
| Lin et al. (2023) | 1 | 1 | 1 | 1 | 0 | 0 | 1 | 0 | 0 | 0 | 0 | 5 |
| Bai et al. (2019) | 1 | 1 | 1 | 1 | 0 | 0 | 1 | 1 | 0 | 1 | 0 | 7 |
| Jing Yan et al. (2021) | 1 | 1 | 1 | 1 | 0 | 0 | 1 | 1 | 0 | 0 | 0 | 6 |
| Zhongjun Wang et al. (2023) | 1 | 1 | 1 | 1 | 0 | 0 | 1 | 1 | 0 | 0 | 0 | 6 |
| Ren et al. (2023) | 1 | 1 | 1 | 1 | 0 | 0 | 1 | 1 | 0 | 1 | 0 | 7 |
| Yingyong Chen et al. b (2022) | 1 | 1 | 1 | 1 | 0 | 0 | 1 | 1 | 0 | 1 | 0 | 7 |
| Xiaowei Wang et al. (2023) | 1 | 1 | 1 | 1 | 0 | 0 | 1 | 1 | 0 | 1 | 0 | 7 |
| Liu et al. (2021) | 1 | 1 | 1 | 1 | 0 | 1 | 1 | 1 | 0 | 1 | 0 | 8 |
| Chengcheng Chen et al. (2023) | 1 | 1 | 1 | 1 | 0 | 0 | 1 | 0 | 0 | 1 | 0 | 6 |
| Wei et al. (2023) | 1 | 1 | 1 | 1 | 0 | 1 | 1 | 0 | 0 | 0 | 0 | 6 |
| Yan Wang et al. (2022) | 1 | 1 | 1 | 1 | 0 | 0 | 1 | 1 | 0 | 0 | 0 | 6 |
| Jiang et al. (2022) | 1 | 1 | 1 | 1 | 0 | 0 | 1 | 1 | 0 | 0 | 0 | 6 |
| Fan et al. (2021) | 1 | 1 | 1 | 1 | 1 | 1 | 0 | 1 | 0 | 1 | 0 | 8 |

Q1:Define the source of information(survey,record review);Q2:List inclusion and exclusion criteria for exposed and unexposed subjects(cases and controls or refer to previous publications);Q3:Indicate time period used for identifying patients;Q4:indicate whether or not subjects were consecutive if not population-based;Q5:Indicated if evaluators of subjective components of study were masked to other aspects of the status of the participants;Q6:Describe any assessments undertaken for assurance purposes;Q7:Explain any patient exclusions from analysis;Q8:Describe how confounding was assessed and/or controlled;Q9:If applicable,explain how missing data were handled in the analysis;Q10:Summarize patient response rated and completeness of data collection;Q11:Clarify what follow-up,if any,was expected and the percentage of patients for which incomplete data or follow-up was obtained.

# Supplementary Figures


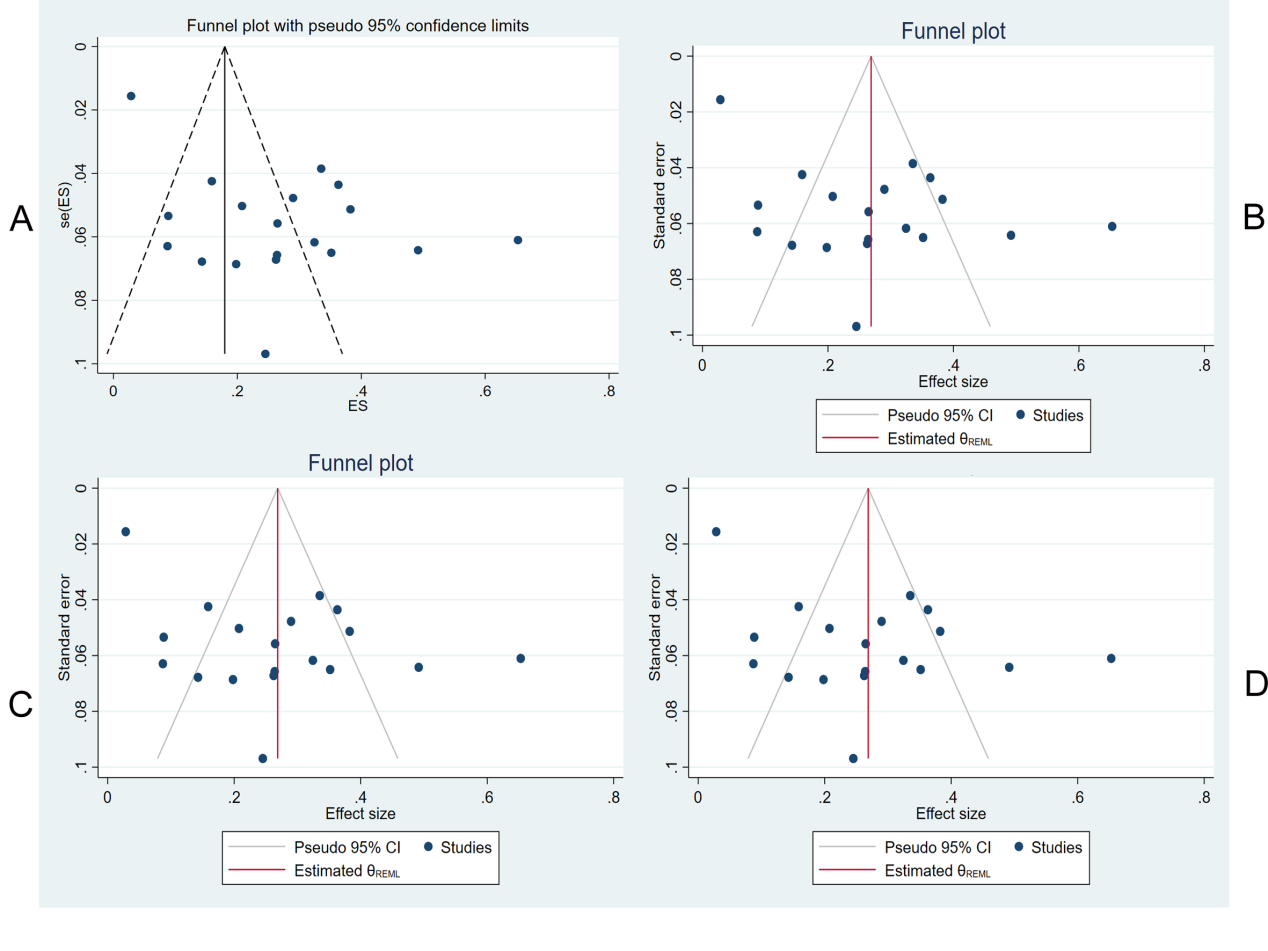


## Supplementary Figure 1 Funnel Plot of Cognitive Frailty Prevalence and Trim-and-Fill Methods: A Funnel Plot, B Trim-and-Fill Run Method, C Trim-and-Fill Quadratic Method, D Trim-and-Fill Linear Method


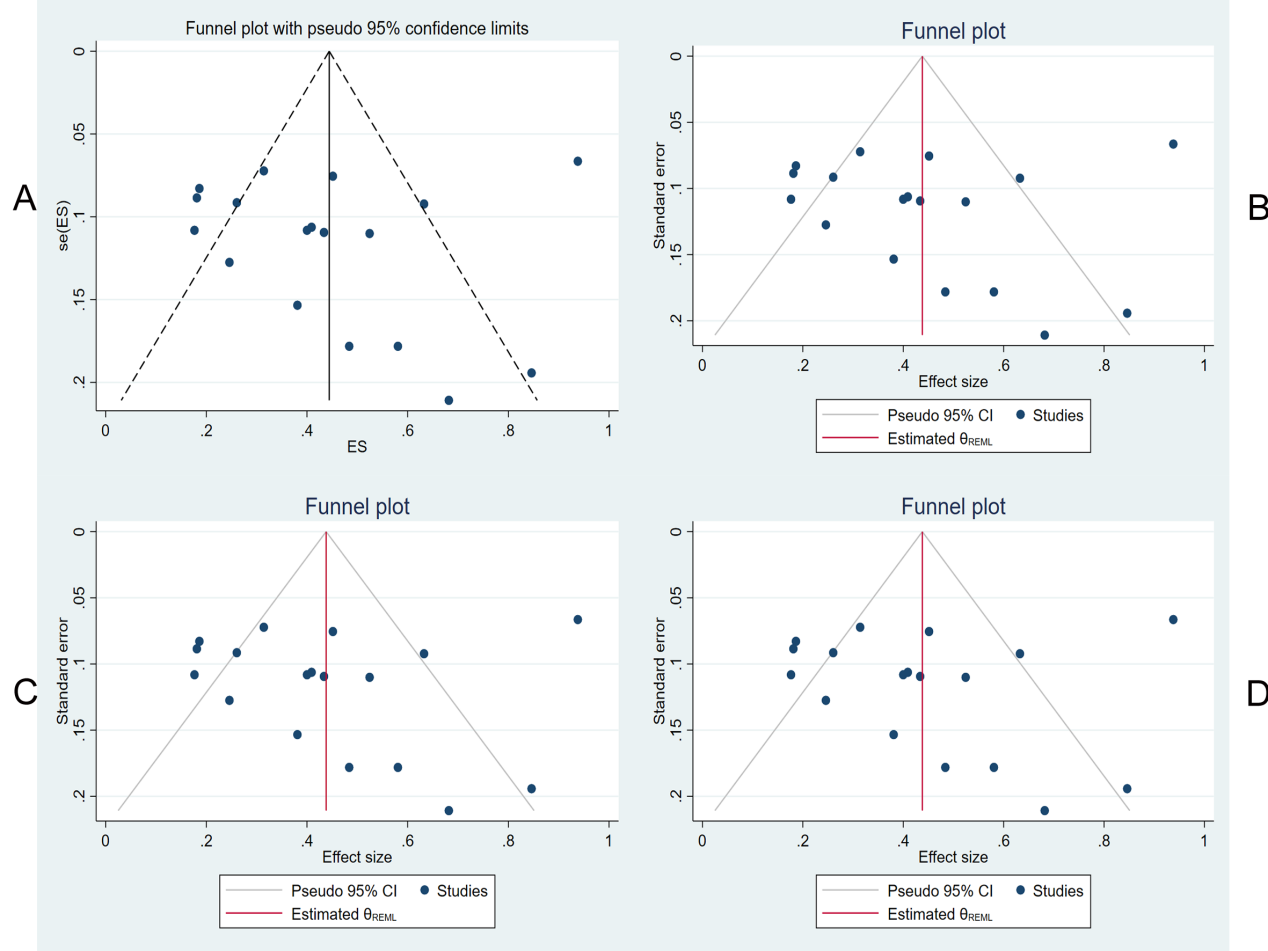


## Supplementary Figure 2 Funnel Plot of Malnutrition Prevalence in Cognitive Frailty and Trim-and-Fill Methods: A Funnel Plot, B Trim-and-Fill Run Method, C Trim-and-Fill Quadratic Method, D Trim-and-Fill Linear Method


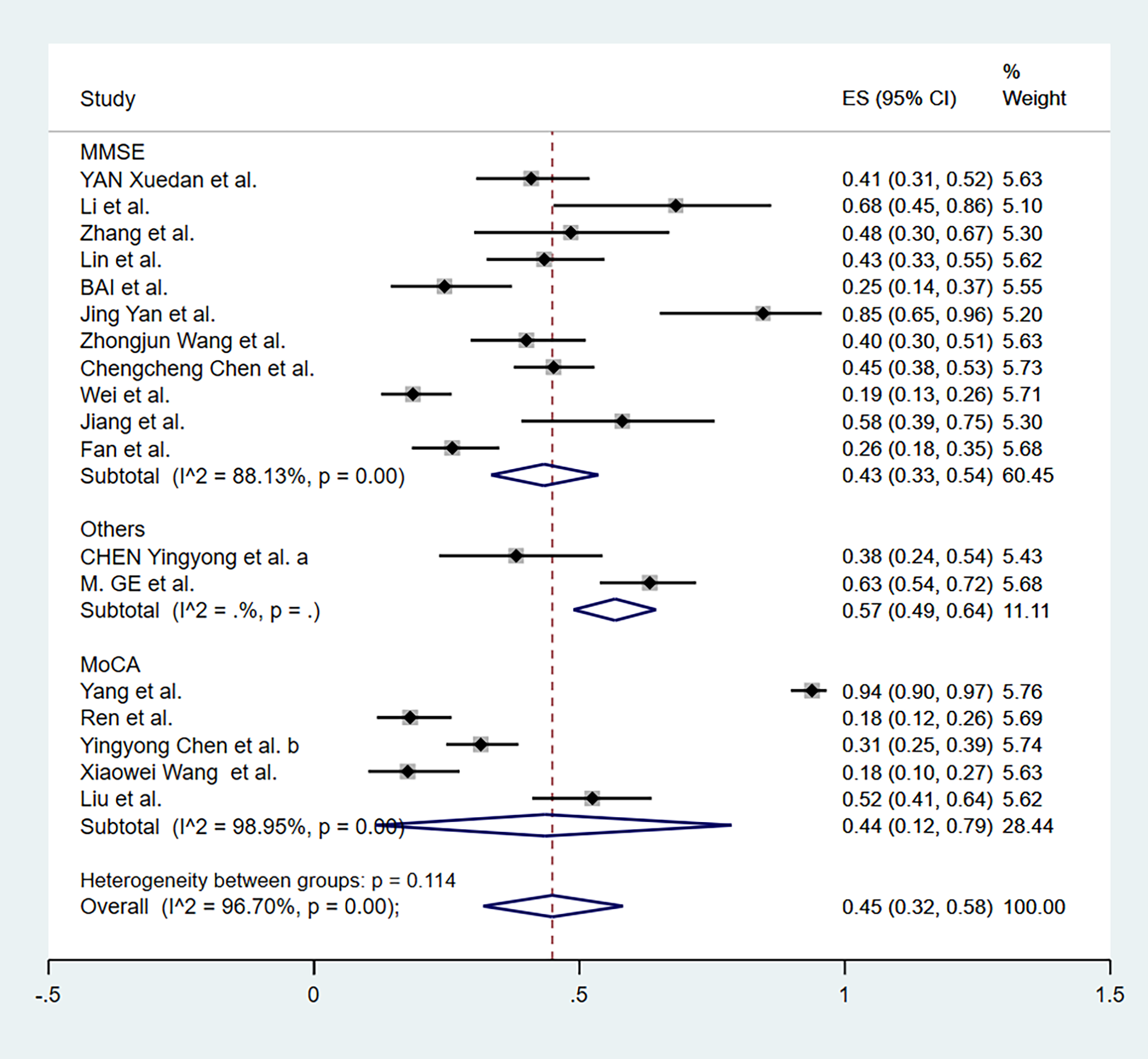


## Supplementary Figure 3 The prevalence of Malnutrition in Cognitive Frailty assessed using different cognitive impairment diagnostic tools


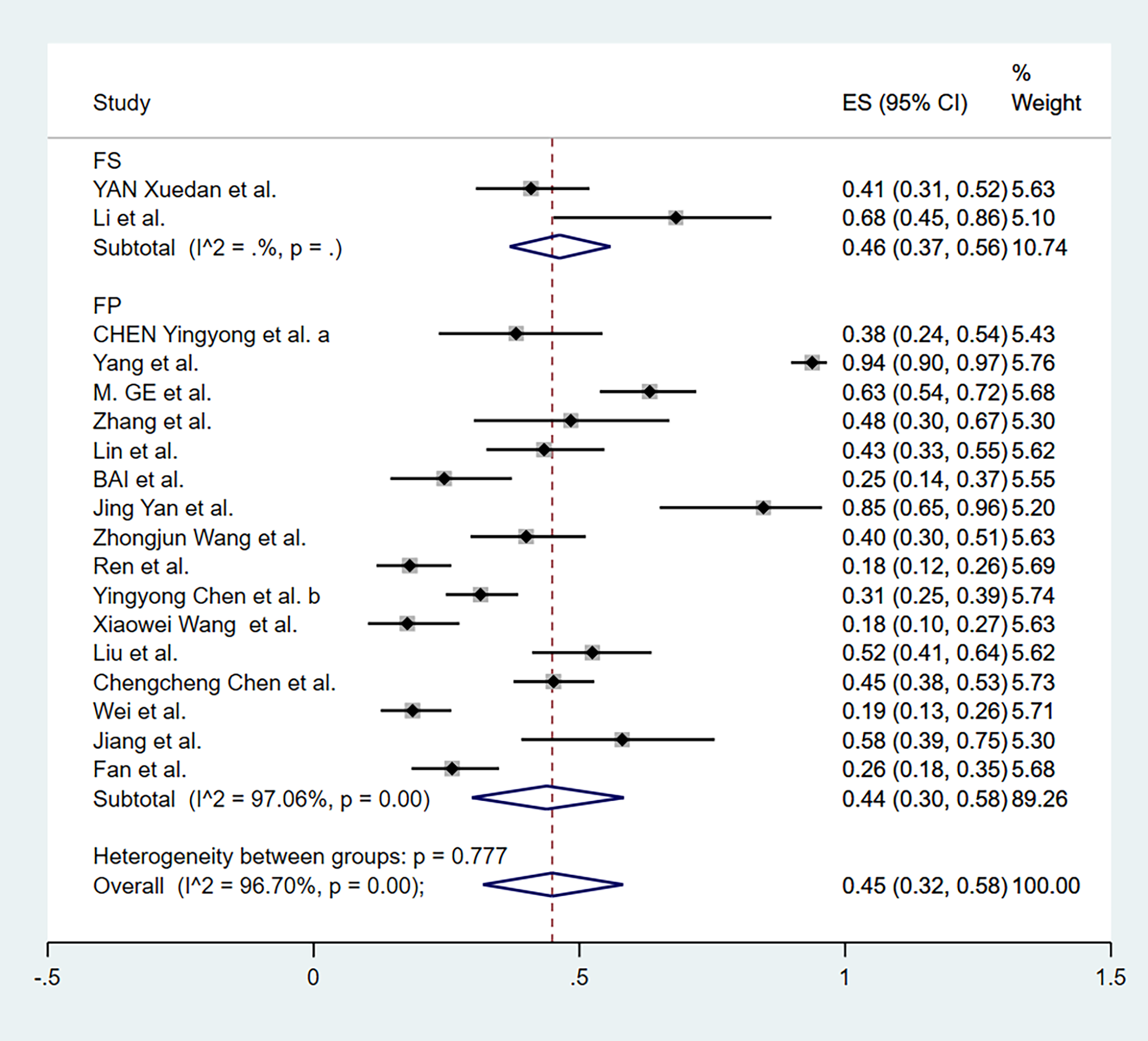


## Supplementary Figure 4 The prevalence of malnutrition in Cognitive Frailty assessed using different frailty diagnostic tools


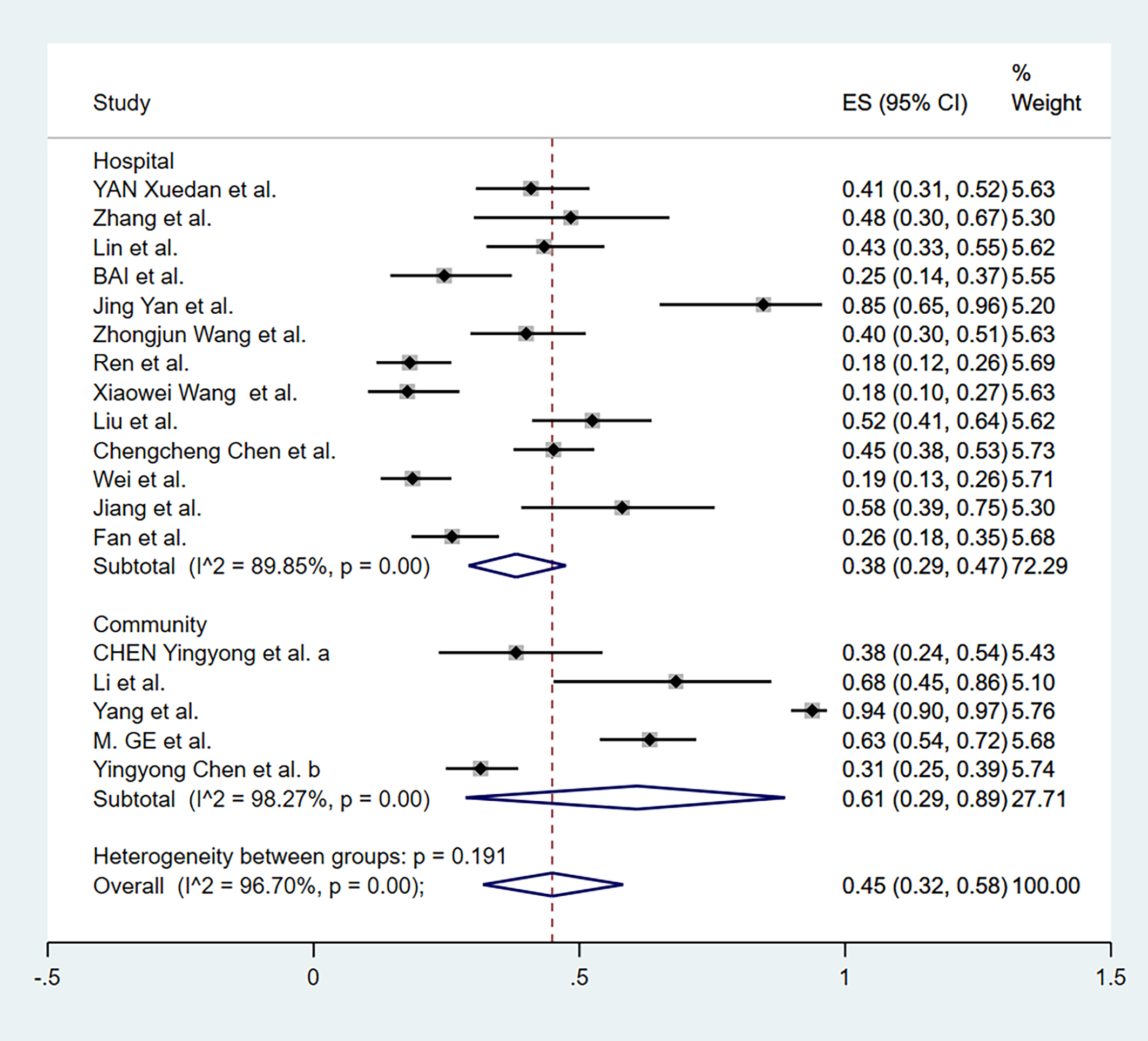


## Supplementary Figure 5 The prevalence of Malnutrition with Cognitive Frailty in different simple source


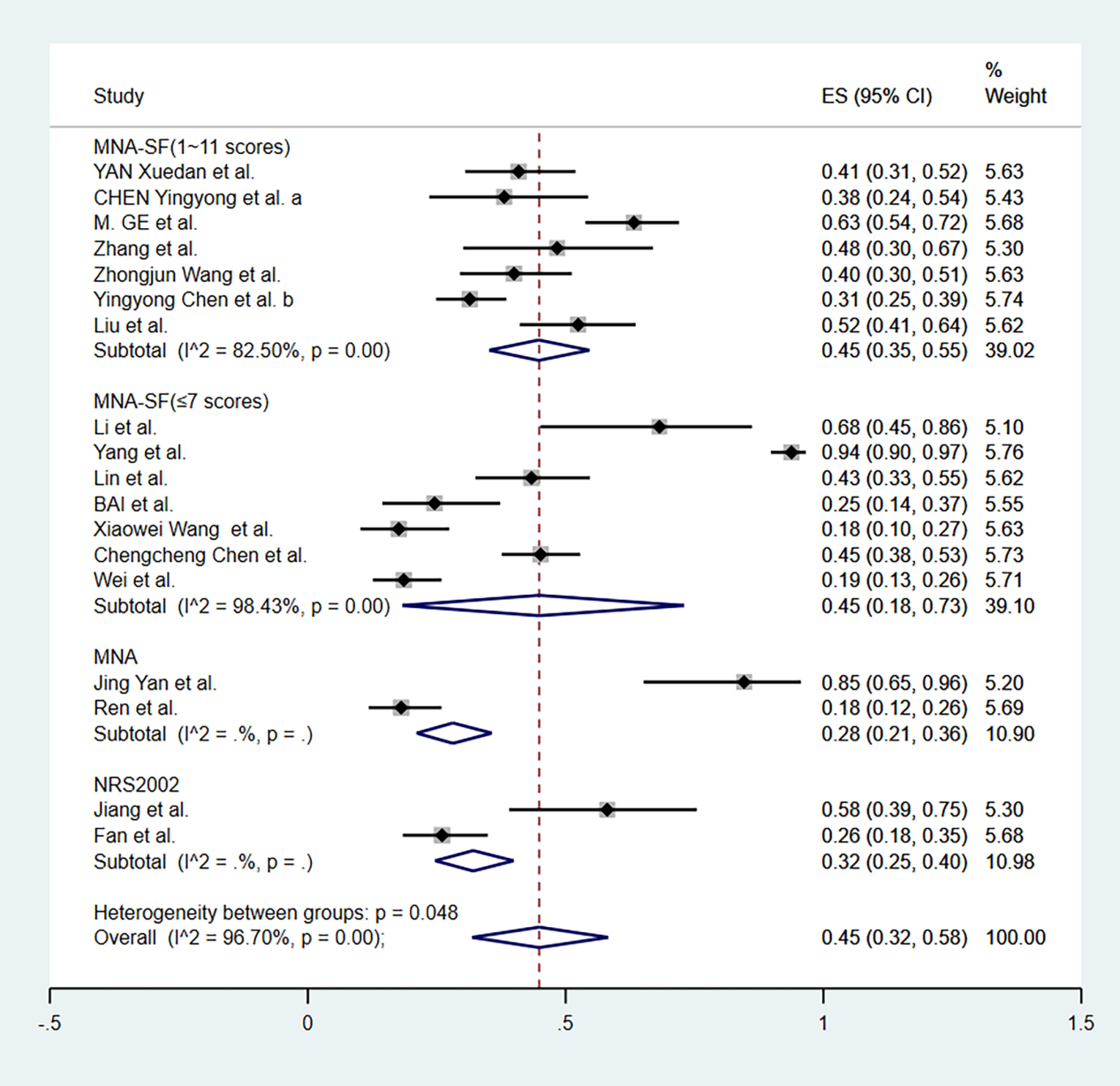


## Supplementary Figure 6 The prevalence of Malnutrition diagnosed using different diagnostic tools among Cognitive Frailty


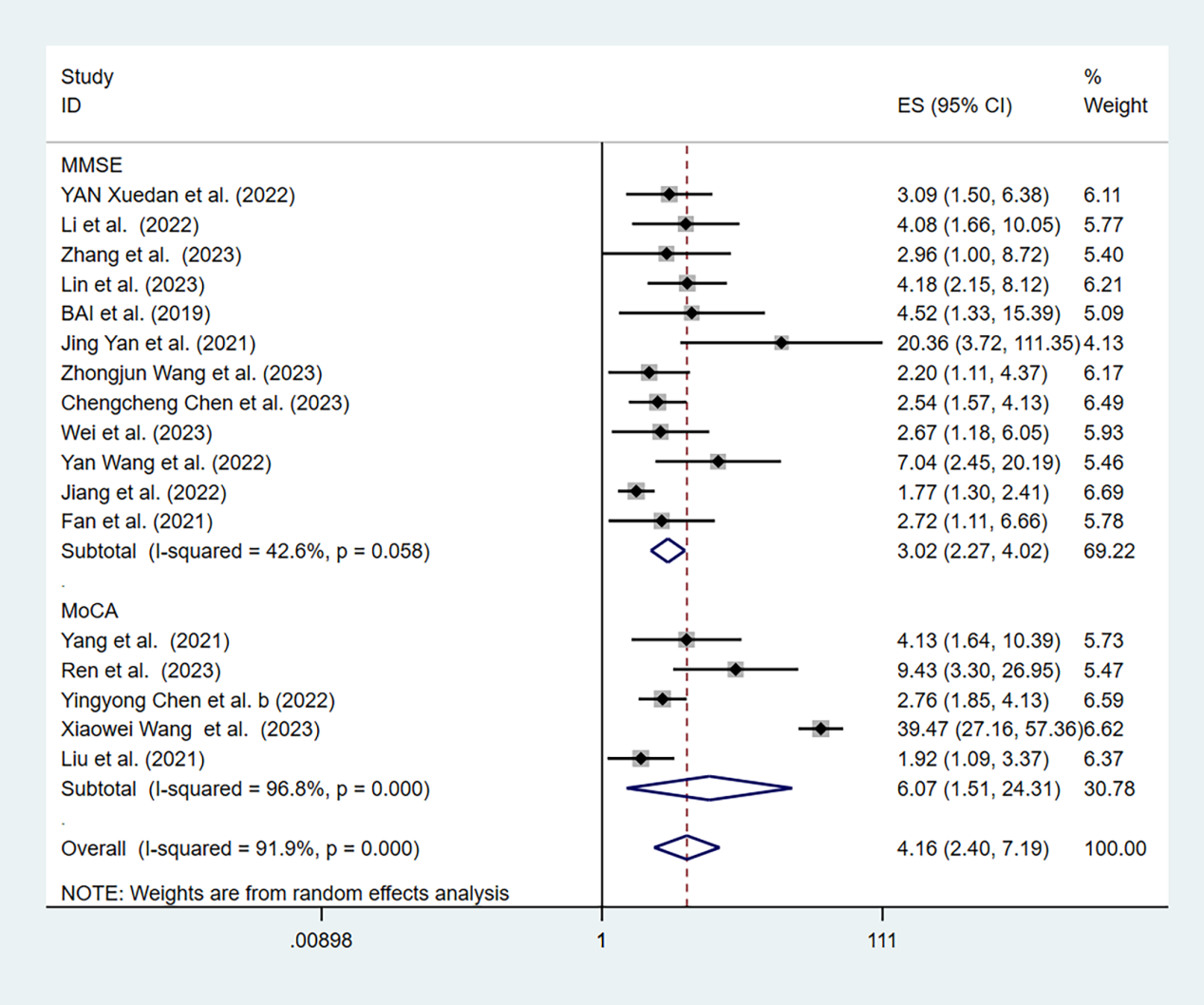


## Supplementary Figure 7 The association between malnutrition and Cognitive Frailty diagnosed using different cognitive function assessment tools


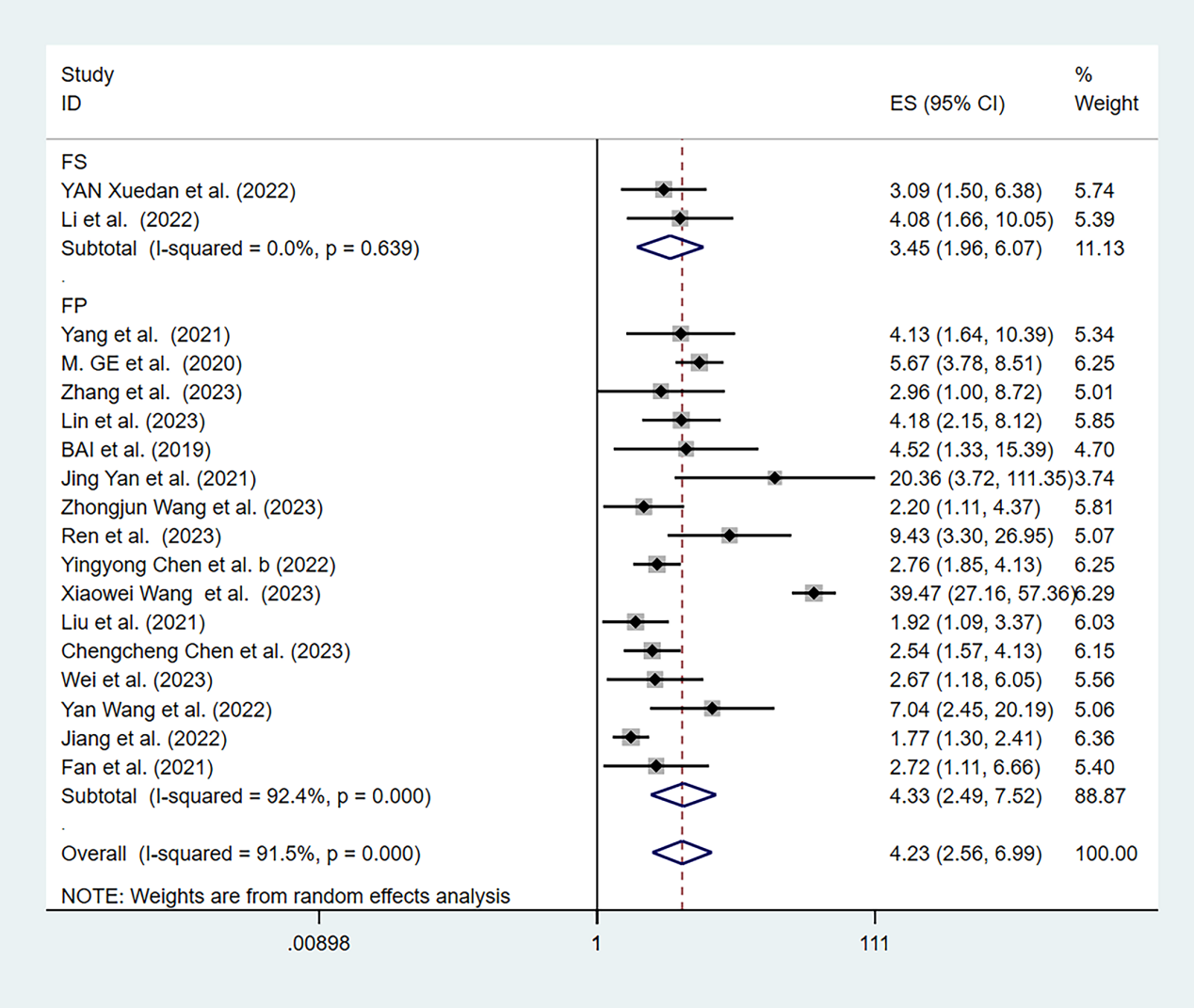


## Supplementary Figure 8 The association between Malnutrition and Cognitive Frailty diagnosed using different frailty assessment tools


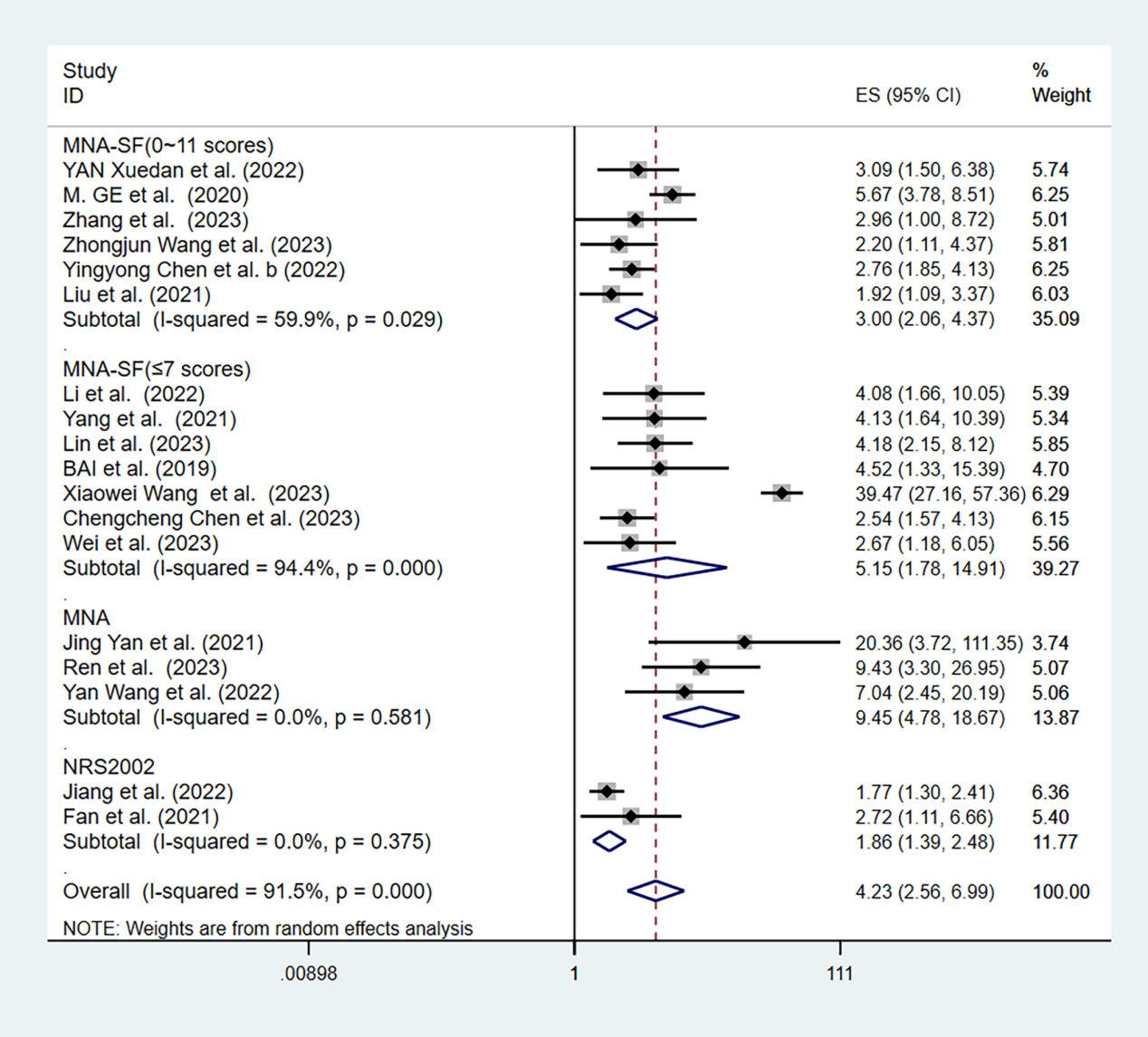


## Supplementary Figure 9 The association between Malnutrition and Cognitive Frailty diagnosed using different Malnutrition assessment tools


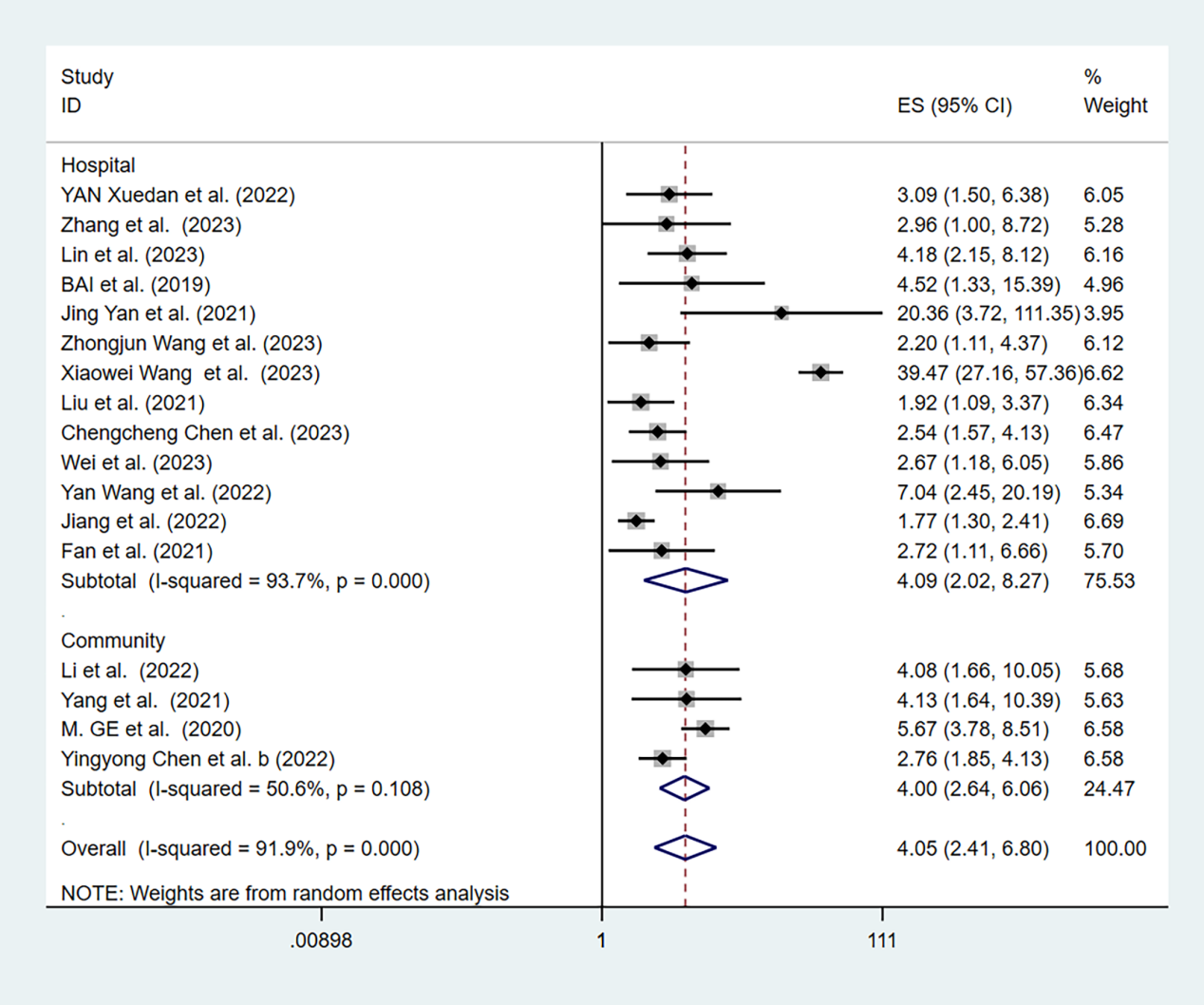


## Supplementary Figure 10 The association between Malnutrition and Cognitive Frailty in different Simple source


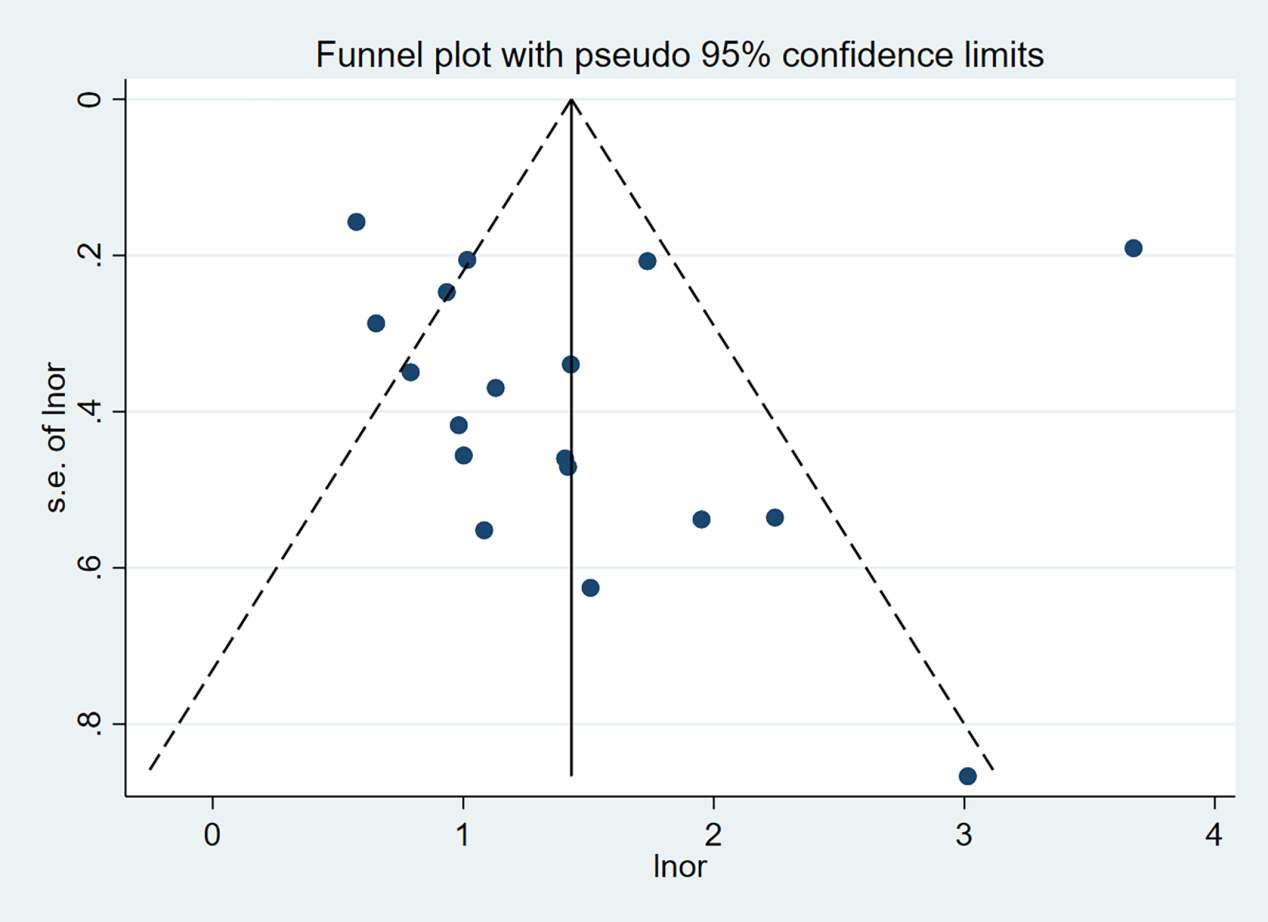


## Supplementary Figure 11 Funnel Plot of the Association Between Cognitive Frailty and Malnutrition
